# SMAD2/3-SMYD2 and developmental transcription factors cooperate with cell-cycle inhibitors to guide tissue formation

**DOI:** 10.1093/procel/pwae031

**Published:** 2024-05-17

**Authors:** Stefania Militi, Reshma Nibhani, Martin Pook, Siim Pauklin

**Affiliations:** Botnar Research Centre, Nuffield Department of Orthopaedics, Rheumatology and Musculoskeletal Sciences, University of Oxford, Old Road, Headington, Oxford OX3 7LD, United Kingdom; Botnar Research Centre, Nuffield Department of Orthopaedics, Rheumatology and Musculoskeletal Sciences, University of Oxford, Old Road, Headington, Oxford OX3 7LD, United Kingdom; Botnar Research Centre, Nuffield Department of Orthopaedics, Rheumatology and Musculoskeletal Sciences, University of Oxford, Old Road, Headington, Oxford OX3 7LD, United Kingdom; Botnar Research Centre, Nuffield Department of Orthopaedics, Rheumatology and Musculoskeletal Sciences, University of Oxford, Old Road, Headington, Oxford OX3 7LD, United Kingdom

**Keywords:** human pluripotent stem cells, epigenetics, TGFβ/ACTIVIN-SMAD2/3, differentiation, cell cycle, EZH2, SMYD2

## Abstract

Tissue formation and organ homeostasis are achieved by precise coordination of proliferation and differentiation of stem cells and progenitors. While deregulation of these processes can result in degenerative disease or cancer, their molecular interplays remain unclear. Here we show that the switch of human pluripotent stem cell (hPSC) self-renewal to differentiation is associated with the induction of distinct cyclin-dependent kinase inhibitors (CDKIs). In hPSCs, Activin/Nodal/TGFβ signaling maintains CDKIs in a poised state via SMAD2/3-NANOG-OCT4-EZH2-SNON transcriptional complex. Upon gradual differentiation, CDKIs are induced by successive transcriptional complexes between SMAD2/3-SMYD2 and developmental regulators such as EOMES, thereby lengthening the G_1_ phase. This, in turn, induces SMAD2/3 transcriptional activity by blocking its linker phosphorylation. Such SMAD2/3-CDKI positive feedback loops drive the exit from pluripotency and stepwise cell-fate specification that could be harnessed for producing cells for therapeutic applications. Our study uncovers fundamental mechanisms of how cell-fate specification is interconnected to cell-cycle dynamics and provides insight into autonomous circuitries governing tissue self-formation.

## Introduction

Coordination of stem–cell proliferation and differentiation is essential for normal development, organ homeostasis, and tissue repair. A direct interplay between cell-cycle progression and differentiation has been suggested in somatic stem cells in the skin, brain, gut, and hematopoietic system (Fuchs, 2009; Lange and Calegari, 2010; Li and Clevers, 2010). Of particular interest, differentiation is often associated with a change in cell-cycle length (Coronado et al., 2013; Savatier et al., 1996), suggesting that mechanisms controlling cell-cycle progression could be involved in cell-fate decisions.

The progression of the cell cycle in mammalian cells is primarily controlled by cyclins and cyclin-dependent kinases (CDKs), which affect key transcriptional regulators such as retinoblastoma protein (pRb). The activity of the Cyclin D/CDK complexes and thus cell proliferation is constrained by cyclin-dependent kinase inhibitors (CDKIs), which are subdivided into two families. The INK4 proteins (p16INK4a, p15INK4b, p18INK4c, and p19INK4d) bind to CDK4 and CDK6 and inhibit their kinase activities by interfering with their association with Cyclin D proteins (Sherr and Roberts, 1999) while the Kip/Cip proteins (p21Cip1, p27Kip1, and p57Kip2) inhibit Cyclin E-CDK2 (Lim and Kaldis, 2013; Sherr and Roberts, 1999). Importantly, the Cyclin D-CDK4/6 complex can also bind to Kip/Cip CDKIs. However, this interaction enhances Cyclin D-CDK4/6 activity since proteins such as p27 appear to limit INK4 CDKIs’ capacity to bind this complex (Besson et al., 2007; Polyak et al., 1994; Toyoshima and Hunter, 1994). Hence, a complex combination of INK4 and KIP/CIP protein regulations determines cell-cycle progression.

Besides their function as tumor suppressors, INK4 and KIP/CIP proteins are known to impact cellular differentiation and organogenesis. Genetic studies in the mouse have demonstrated that *Ink4* and *Kip*/*Cip* genes are required for normal development (Buchold et al., 2007; Cunningham et al., 2002; Dyer and Cepko, 2001; Zindy et al., 2001). For example, the absence of *p18* causes widespread organomegaly in mice (Franklin et al., 1998) while mice lacking *p57* die at birth and have multiple developmental abnormalities including hyperplasia and delayed differentiation (Yan et al., 1997; Zhang et al., 1997). On the other hand, *p27* is necessary for normal differentiation of neural stem cells (NSCs) and mESCs (Li et al., 2012; Marques-Torrejon et al., 2013). CDKIs are also essential for stem-cell differentiation in adult organs. *p21* can limit hair follicle quiescence while *p27* has a key function in neuronal differentiation in the cortex (Godin et al., 2012; Nguyen et al., 2006). Considered collectively, these reports suggest that *CDKIs* have a function during stem cell and progenitor cell-fate specification beyond their tumor suppressive functions. However, the precise interplay between signals of differentiation and transcriptional networks regulating CDKI expression remains to be fully elucidated.

Human pluripotent stem cells (hPSCs) represent a unique model system to study such mechanisms. These pluripotent cells can self-renew indefinitely while maintaining the capacity to differentiate into the three primary germ layers neuroectoderm, mesoderm, and endoderm. Accordingly, hPSCs have been used by multiple groups to study the mechanisms by which cell-cycle control differentiation (Calder et al., 2012; Gonzales et al., 2015; Pauklin and Vallier, 2013; Singh et al., 2013). Here we took advantage of this model system to investigate the mechanisms by which CDKIs can coordinate cell cycle and differentiation. We found that INK4 and KIP/CIP display a tissue-specific expression during hPSC differentiation and that their induction is controlled by a distinct set of developmental signaling pathways including ACTIVIN/NODAL-SMAD2/3. We uncovered that ACTIVIN/NODAL establishes a characteristic CDKI expression pattern in hPSCs via an activating NANOG-SMAD2/3 complex on *p27* locus and instead a transcriptionally repressive NANOG-SMAD2/3-SNON complex on other *CDKI* loci. These transcriptional complexes are substituted during the exit from the stem-cell self-renewal state and entry into cell-fate specification. The induction of CDKI proteins upon differentiation via developmental master regulators such as the EOMES-SMAD2/3 complex results in an increase of the G_1_ phase length especially during endoderm specification. This extended G_1_ phase enables SMAD2/3 to accumulate into the nucleus of cells differentiating toward endoderm and allowing the transcriptional circuitry associated with ACTIVIN/NODAL signaling to be strongly enhanced. These results reveal positive feedback loops between developmental transcription factors and CDKIs (p15/p18/p27/p57), which dynamically guide the specification of stem cells along different lineages. Collectively, our results unravel the molecular mechanisms by which cell-fate decisions are controlled by the cell-cycle machinery and describe a dynamical coordination of lineage specification with cell-cycle progression.

## Results

### CDKIs are induced during hPSC differentiation and display distinct expression patters in germ layers

**Figure 1.**
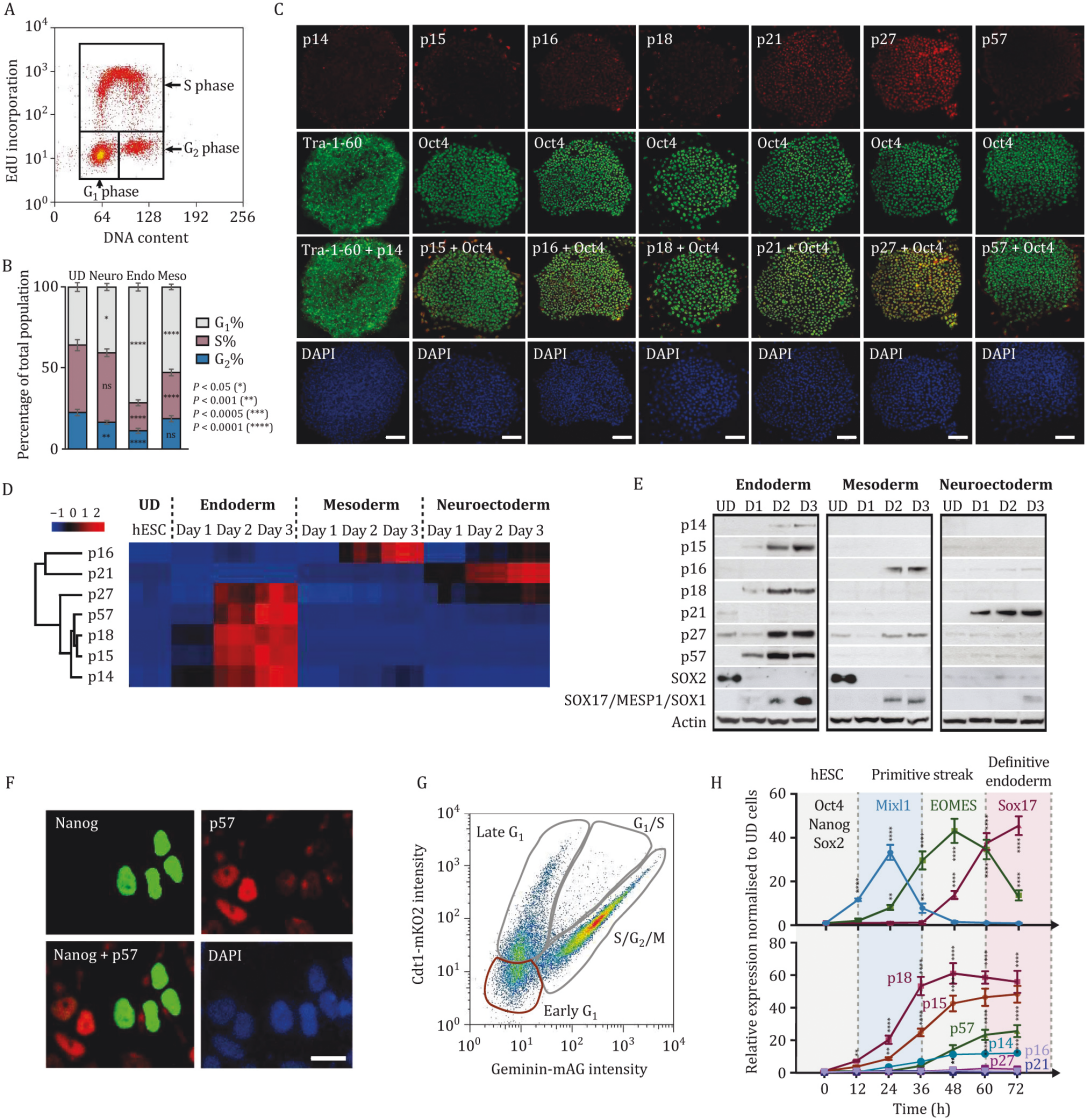
CDKIs are not expressed in self-renewing hPSCs while rapidly induced during germ layer differentiation with simultaneous changes in cell-cycle length. (A) Representative dot blot graph of EdU incorporation and DNA content analysis for determining the cell-cycle profile. (B) Endoderm cells have a strongly lengthened G_1_ phase compared to other germ layers. Cell-cycle analysis by EdU incorporation during germ layer differentiation by collecting cells 72 h after initiating endoderm, mesoderm, or neuroectoderm differentiation. (C) CDKIs p14, p15, p16, p18, p21, and p57 are not expressed in hPSCs except for p27 protein. Immunostaining of CDKIs together with pluripotency markers Tra-1-60 or OCT4. CDKI expression is not detectable except for p27 protein in undifferentiated hPSCs. Scale bar, 100 μm. (D and E) CDKIs are specifically induced during the germ layer formation. Relative changes in CDKI (D) mRNA by Euclidian hierarchical clustering or (E) protein compared to undifferentiated hPSCs. Z-scores in the heat map indicate the differential expression measured in number of standard deviations from the average level across all conditions. In Western blot we used the following germ layer markers: SOX17 for endoderm, MESP1 for mesoderm, and SOX1 for neuroectoderm. (F) CDKIs accumulate in the nucleus upon their induction in differentiating cells. Immunostaining of p57 and NANOG in a mix of pluripotent and differentiating cells at 36 h of endoderm differentiation. Scale bar, 10 μm. (G) Dot blot graph of unsynchronised FUCCI-hPSCs visualized by flow cytometry. The different cell-cycle phases are marked with gray lines, and early G_1_ phase cells sorted are marked with red lines. (H) CDKI induction is an early event during the initiation of differentiation. Dynamical changes of endoderm gene expression and CDKIs marks on synchronized cells differentiated toward definitive endoderm and analysed by Q-PCR. Statistical analysis was performed by 2-way ANOVA with multiple comparisons with Tukey correction and **** marks adjusted *P*-value < 0.0001, *** is adjusted *P*-value < 0.001, ** is adjusted *P*-value < 0.01, * is adjusted *P*-value < 0.05.

To explore the interplays between cell-cycle progression and cell-fate decisions, we decided to first, characterize the regulation of CDKIs in pluripotent self-renewing hPSCs and during their differentiation. We determined the cell cycle profile of hPSCs differentiating toward endoderm, mesoderm, and neuroectoderm by EdU incorporation analyses, which revealed that differentiation systematically results in an increase of the G_1_ phase length. However, this increase was markedly different between each differentiated cell type (Fig. 1A and 1B). Lengthening of G_1_ phase remained limited in neuroectoderm while being considerably increased in the endoderm lineage. To find out if any cells are entering the G_0_ phase in endoderm differentiation, we generated H9 hPSCs with a three-color FUCCI system, which allows for the detection of cells in early G_1_, late G_1_, G_1_/S transition, S/G_2_/M, and G_0_ phases. Differentiation of these H9-FUCCI cells to the three germ layers indicated that cells only increase in G_0_ phase in endoderm differentiation whereas neuroectoderm and mesoderm cells do not increase cell fraction in G_0_ phase (Fig. S1A). Collectively, these data support previous reports (Calder et al., 2012; Coronado et al., 2013; Savatier et al., 1996), showing that differentiation is associated with a change in cell cycle. It also suggests that factors controlling the length of G_1_ phase could be induced upon differentiation of hPSCs.

To further validate this hypothesis, we investigated the expression pattern of CDKIs during hPSC differentiation since these proteins represent the main inhibitors of cell cycle progression. Immunostaining, Q-PCR, and Western blot analyses revealed that most of the CDKIs (p14, p15, p16, p18, and p57) are not expressed in hPSCs with the exception of p27 in some cells, and to a lesser extent p21 (Fig. 1C–G). Similar analyses revealed that CDKIs display distinct expression patterns during germ layer specification (Figs. 1D, 1E and S1B–D). p18, p15, p14, p27, and p57 showed a specific induction during endoderm specification while mesoderm differentiation was accompanied by an induction of p27 and p16. Finally, only p21 was significantly increased during neuroectoderm specification (Figs. 1D, 1E and S1B). Further coexpression analyses of OCT4 or NANOG with CDKIs indicate the general non-overlapping expression of these pluripotency factors with CDKIs in early endoderm differentiation at Day 1, except p27 (Fig. S1C and S1D). We also analyzed the expression of CDKIs by IF during germ layer differentiation. As expected, the positive control of differentiation indicated the loss of pluripotency factor SOX2, OCT4, and NANOG expression during germ layer differentiation at 72 h, except for SOX2, which has a high expression in neuroectoderm (Fig. S1E). Full IF analyses during endoderm at 72 h (Fig. S2A), mesoderm at 72 h (Fig. S2B), and neuroectoderm differentiation at 72 h (Fig. S2C), provided detailed insight into CDKI protein induction at the single-cell level and showed most extensive expression of CDKIs in endoderm germ layer. Endoderm cells at Day 3 were also used for co-immunostaining for CDKIs and the marker of proliferation ki67, which indicated that ki67 signal is present in cells that do not express CDKIs (Fig. S3A). Together these data suggest that self-renewing pluripotent stem cells keep most of the CDKIs in a repressed state and avoid their expression, while CDKIs could have a tissue-specific function during hPSC differentiation.

Next, we analyzed the precise timing of CDKI induction upon endoderm differentiation of hPSCs since the specification of this germ layer is associated with the upregulation of several CDKIs. We took advantage of FUCCI-hPSCs since these cells can be synchronized in different phases of the cell cycle by cell sorting (Pauklin and Vallier, 2013). In this case, FUCCI-hPSCs were synchronized to early G1 (Fig. 1G) and then differentiated to endoderm. This approach results in a population of cells, which more homogenously differentiate to endoderm while progressing with the same timing through the cell cycle (Madrigal et al., 2023). hPSCs differentiating from endoderm using this approach were examined for the expression of mesendoderm/endoderm and CDKI markers every 6 h (Fig. 1H and Supplementary Information). These analyses revealed that CDKIs are induced concomitantly with early mesendoderm markers such as MIXL1 prior to the induction of the definitive endoderm marker SOX17. This very rapid induction could enable CDKIs to inhibit cyclin/CDK complexes during cell-cycle progression upon differentiation thereby explaining the lengthening of the G_1_ phase characterizing the cell-cycle profile of endoderm cells.

Altogether, these results suggest that CDKI expression in tightly controlled in pluripotent stem cells and tissue-specific induction of CDKIs determines the cell-cycle profile of hPSC derivatives after differentiation into the three germ layers.

### A compound library screen of epigenetic inhibitors identifies EZH2 and SMYD2 as factors governing hPSC self-renewal and differentiation

To uncover novel epigenetic regulators of hPSCs, we used three pluripotency markers (OCT4, CD133, SSEA4) for identifying pluripotent and differentiated cells in the experiments. The H9 hPSCs used for this screening were genetically engineered, with the sequence coding for green fluorescent protein (eGFP) integrated with the endogenous locus, resulting in controlled expression of a OCT4-eGFP fusion protein driven by the endogenous *OCT4* promoter. The CD133 and SSEA4 are widely used pluripotency markers expressed in hPSCs but lost in differentiated hPSCs.

**Figure 2.**
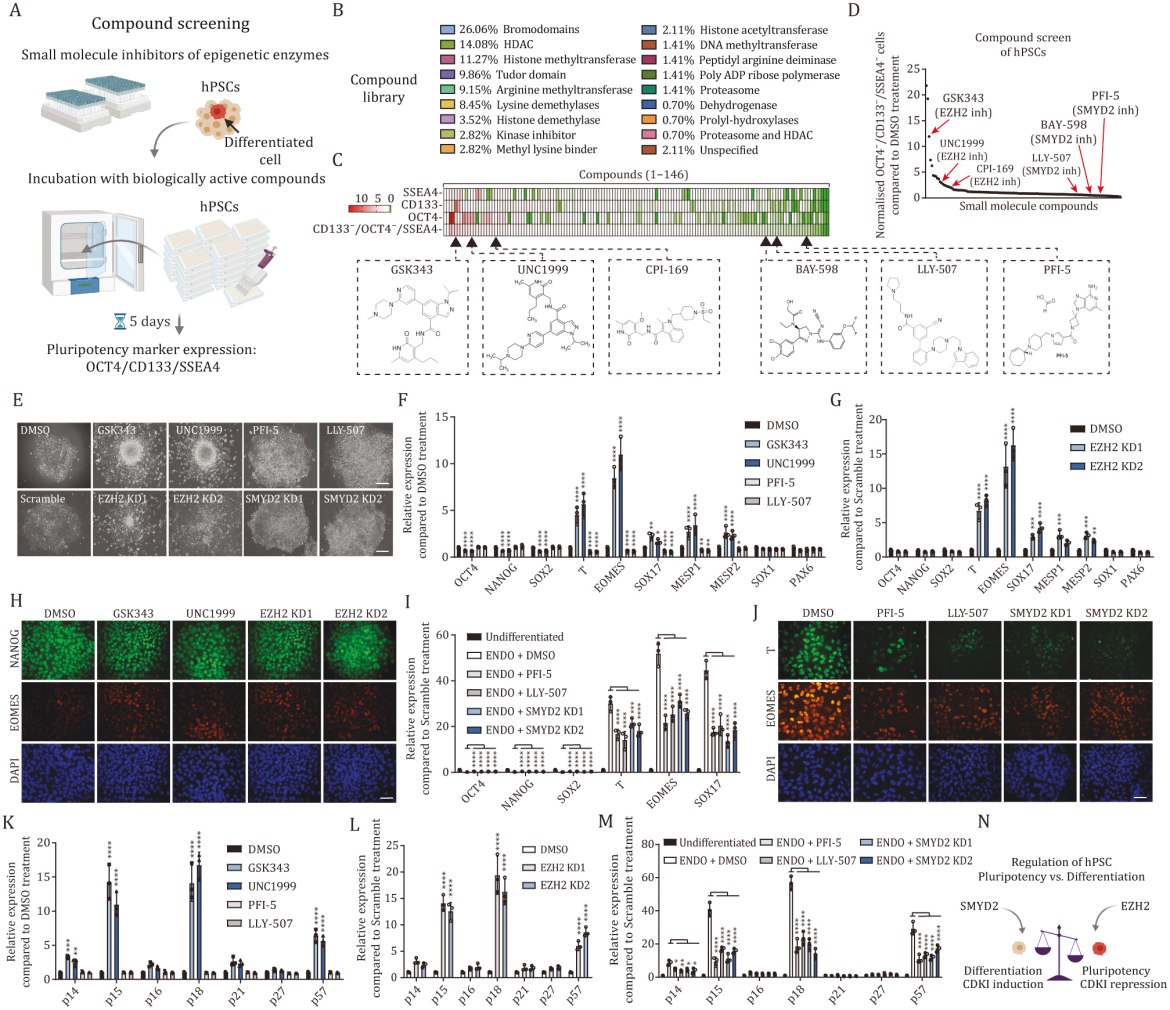
Compound screening identifies SMYD2 and EZH2 as regulators of hPSC self-renewal and differentiation. (A) Schematic representation of the small molecule screening process on hPSCs. (B) Compounds in the epigenetic library. (C and D) EZH2 inhibitors GSK343, UNC1999, and CPI-169 increase the fraction of pluripotent marker negative cells whereas SMYD2 inhibitors PFI-5, LLY-507, and BAY-598 decrease pluripotent marker negative cells compared to DMSO control treatment. (E) Representative colonies of hPSCs treated with EZH2 inhibitors, SMYD2 inhibitors, or EZH2 and SMYD2 KD. (F) Treatment of hPSCs with EZH2 inhibitors increases endoderm and mesoderm marker expression. (G) EZH2 KD in hPSCs increases endoderm marker expression. (H) Immunostaining of EOMES and NANOG in EZH2 inhibitor treated hPSCs or EZH2 KD cells. (I) SMYD2 inhibition and knockdown reduce endoderm marker expression upon endoderm differentiation at 36 h timepoint. (J) Immunostaining of EOMES and T in hPSCs differentiated to endoderm for 36 h and treated with SMYD2 inhibitors or SMYD2 KD. (K) Several CDKIs are upregulated upon EZH2 inhibition in hPSCs. (L) EZH2 KD upregulates p15, p18, and p57 expression in hPSCs. (M) SMYD2 chemical inhibition and genetic knockdown downregulates several CDKIs during endoderm differentiation at 36 h. (N) Schematic depiction of the effects of SMYD2 and EZH2 on pluripotency and differentiation in hPSCs. Statistical analysis was performed by two-way ANOVA with multiple comparisons with Tukey correction and **** marks adjusted *P*-value < 0.0001, *** is adjusted *P*-value < 0.001, ** is adjusted *P*-value < 0.01, * is adjusted *P*-value < 0.05.

We hypothesized that the self-renewal of hPSCs and the early cell-fate decisions of hPSCs leading to CDKI expression during germ layer differentiation are controlled by epigenetic mechanisms. To address this, we performed a focused library screen consisting of validated small molecule inhibitors (Table S4) targeting epigenetic regulators such as “readers, writers, and erasers” of a histone code (Hyun et al., 2017). These experiments aimed to identify molecular targets of small molecule compounds that specifically inhibit epigenetic regulators and thereby affect pluripotency marker-expressing cells (Fig. 2A). We measured pluripotency marker (CD133, OCT4, SSEA4) expression by flow cytometry and hPSC cell growth after incubating the cells with the compounds for 5 days, which allowed us to identify effective compounds that impact pluripotency marker expressing cells while also detecting cells that do not express these markers which represent differentiated hPSCs. The compound library used in our experiments consisted of 142 compounds that have been verified to be active and targeting specific epigenetic modifying enzymes (Fig. 2B). Overall, this screening identified compounds that significantly and reliably reduced the relative percentage of marker positive hPSCs and negative (CD133^−^/OCT4^−^/SSEA4^−^) differentiated hPSCs (Fig. 2C and 2D).

The screening identified novel compounds that target distinct epigenetic regulatory components and altered the percentage of pluripotent cells versus differentiated cells. Among the top candidate compounds that increased differentiation evidenced by increased pluripotency marker negative cells were EZH2 inhibitors (GSK343, UNC1999, CPI-169) (Fig. 2C and 2D). In contrast, among the top candidate compounds that decreased background differentiation evidenced by decreased marker negative cells were SMYD2 inhibitors (PFI-5, LLY-507, BAY-598) (Fig. 2C and 2D). These results suggested that EZH2 inhibition could induce differentiation of hPSCs whereas SMYD2 inhibition could block differentiation of hPSCs. EZH2 is a subunit of polycomb repressive complex 2 (PRC2), which catalyzes the trimethylation of H3K27 at developmental gene promoters (Cao et al., 2002). This maintains the transcriptionally repressive state of genes associated with differentiation to retain stem-cell pluripotency in hPSCs (Boyer et al., 2006). The SMYD (SET and MYND domain-containing protein) family contains five members (SMYD1–5) that share a catalytic SET domain and an MYND motif, which are involved in lysine methylation and protein–protein interaction, respectively. SMYD2 has been reported to suppress cell proliferation by mediating H3K36me2 deposition (Brown et al., 2006), and in transcriptional regulation by mediating H3K4me1 (Abu-Farha et al., 2008). In addition, SMYD2 is reported to methylate the K370 site of p53 repressing its antitumor effect (Huang et al., 2006), whereas SMYD2 induces RB1 methylation at lysine 810 in some cancers that promotes cell cycle progression of these malignant cells (Cho et al., 2012).

To further validate EZH2 and SMYD2 as a target we used two EZH2 chemical inhibitors (GSK343 and UNC1999) and two SMYD2 chemical inhibitors (PFI-5 and LLY-507) to investigate their effects on hPSCs (Fig. 2E and 2F). Treatment of hPSCs with EZH2 inhibitors indicated a loss of pluripotency and increased differentiation based on cell morphology (Fig. 2E), while qPCR analyses revealed a reduction of core pluripotency factors OCT4, NANOG, and SOX2 with concomitant differentiation mostly to endoderm (T, EOMES, SOX17) and less to mesoderm or primitive streak (MESP1, MESP2) but not to neuroectoderm (SOX1, PAX6) (Fig. 2F and 2H). We also observed increased differentiation to endoderm and less to mesoderm by qPCR analyses of EZH2 knockdown hPSCs (Figs. 2E, 2G, 2H and S3B). In contrast, SMYD2 inhibitors did not increase the differentiated morphology of hPSCs and did not increase spontaneous differentiation of hPSCs to germ layers but had a consistently lower background signal of endoderm markers (Figs. 2E, 2F and S3B). Therefore, we investigated SMYD2 knockdown effects upon endoderm differentiation. We found that cells differentiated to endoderm for 36 h with SMYD2 inhibitors indicated higher expression of pluripotency markers OCT4, NANOG, and SOX2 while there is a reduced expression of T, EOMES, and SOX17 compared to control endoderm differentiation (Fig. 2I and 2J). SMYD2 knockdown also reduced differentiation to endoderm as shown by qPCR analyses and immunofluorescence microscopy of SMYD2 knockdown (Fig. 2I and 2J).

Next, we hypothesized that EZH2 and SMYD2 could regulate the expression of CDKIs in hPSCs and during endoderm differentiation. To investigate this, we analyzed the expression of CDKIs upon the treatment with EZH2 and SMYD2 inhibitors. EZH2 inhibition resulted in the induction of p15, p18, and p57 in hPSCs upon both chemical inhibition and genetic depletion of EZH2 (Fig. 2K and 2L). In contrast, SMYD2 inhibition did not upregulate CDKI expression in hPSCs (Fig. 2K), but SMYD2 chemical inhibition and genetic depletion both attenuated the induction of CDKIs during endoderm differentiation (Fig. 2M).

Since EZH2 and SMYD2 are acknowledged as epigenetic regulators, we performed H3K27me3 ChIP-qPCR analyses of hESCs at the CDKI loci upon the inhibition of EZH2 with GSK343 or UNC1999. The inhibition of EZH2 led to the reduction of H3K27me3 modification at key endoderm (T, EOMES, SOX17), mesoderm (MESP1, MESP2) and neuroectoderm (SOX1) loci (Fig. S3C), whereas we observed also a reduction on CDKI loci p14/p16, p15, p18, p21, and p57 (Fig. S3D). This suggested that both developmental loci of germ layers as well as CDKI loci are regulated by EZH2 in hESCs that mediate the deposition of the H3K27me3 repressive mark in undifferentiated conditions. Next, we performed H3K4me3 ChIP-qPCR analyses of differentiating hESCs at the CDKI loci upon the inhibition of EZH2 with PFI-5 or BAY-598. The inhibition of SMYD2 resulted in the decrease of H3K4me3 histone modification at endoderm (T, EOMES, SOX17), mesoderm (MESP1, MESP2), and neuroectoderm (SOX1, PAX6) loci (Fig. S3E). Furthermore, we observed a reduction of H3K4me3 modification on CDKI loci (Fig. S3F), indicating that SMYD2 deposits H3K4me3 modification during hPSC differentiation onto germ layer loci but also CDKI loci. Therefore, SMYD2-mediated H3K4me3 modification contributes to gene expression regulation during germ layer formation.

Collectively, our compound screening identified EZH2 and SMYD2 as epigenetic factors regulating hPSC self-renewal and endoderm differentiation that also govern CDKI expression in hPSCs and early differentiation to endoderm (Fig. 2N).

### Developmental signaling pathways control the expression of different CDKIs during cell-fate specification

The differential expression of CDKIs in germ layers indicated that their expression could be controlled by different sets of signaling pathways. To investigate this hypothesis, we blocked individually each pathway (ACTIVIN A/TGFβ, BMP4, FGF2, PI3K, WNT) known to be involved in endoderm specification and then measured the expression of each CDKI by qPCR. Inhibition of ACTIVIN/NODAL signaling resulted in the loss of p15, p18, p27, and p57 expression, while FGF2 inhibition decreased p18 expression and BMP4 inhibition reduced p15 and p14 induction (Fig. S3G) while increased ACTIVIN and BMP4 induced these CDKIs, respectively (Fig. S3H and S3I). The absence of Ly-294002 that allows for PI3K activity caused a loss of p15 and p57 expression while increasing p18 expression (Fig. S3G). WNT pathway activation with CHIR99021 during hPSC differentiation impacted the expression of p14, p18, p21, and p27 (Fig. S3G). Therefore, developmental pathways directing cell-fate choice also appear to control the expression of CDKIs, uncovering a crosstalk between differentiation signals and cell-cycle regulation.

**Figure 3.**
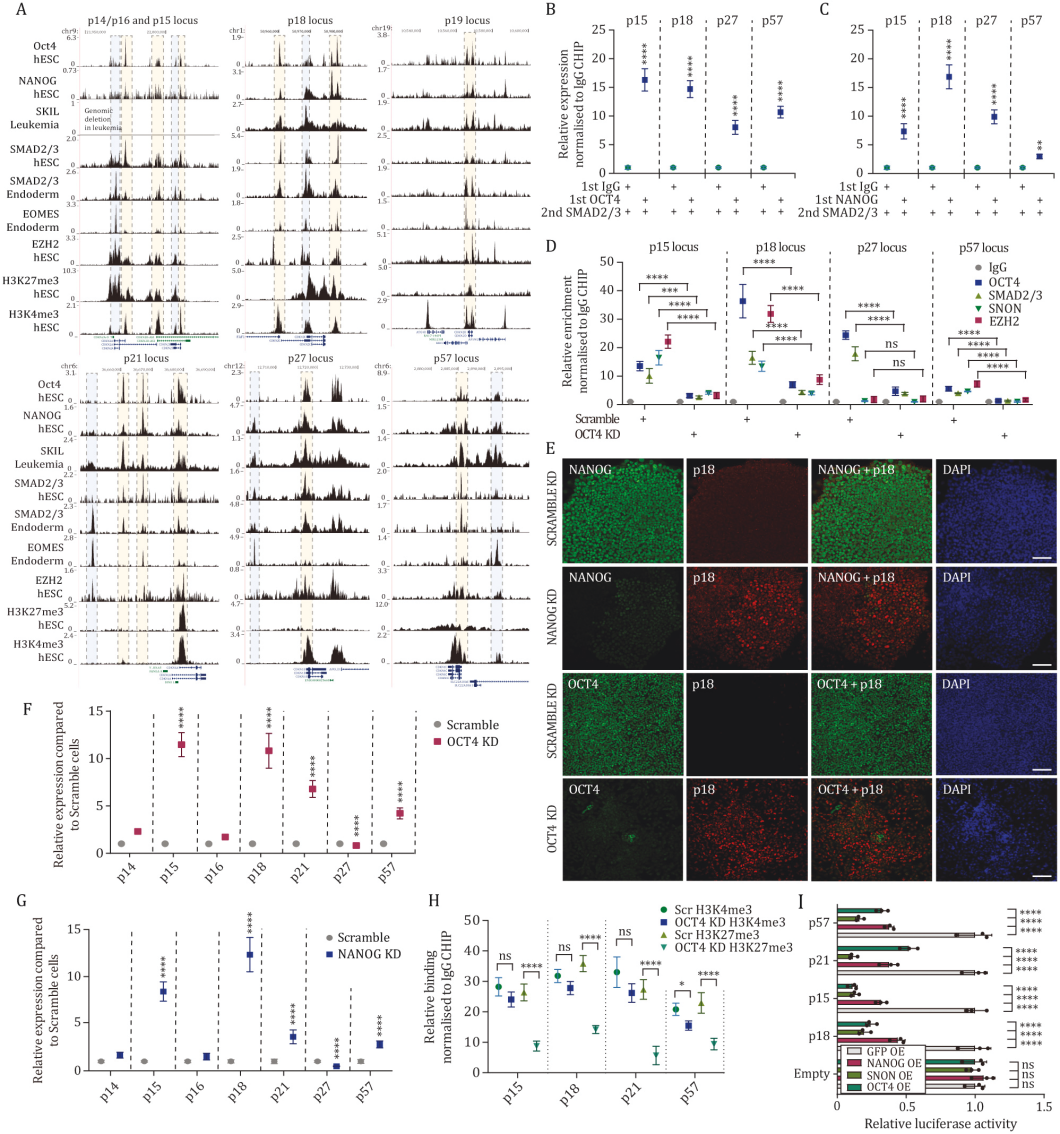
ACTIVIN/NODAL signaling establishes a stem cell-specific expression pattern of CDKIs with poised chromatin through cooperation between NANOG/OCT4, SMAD2/3, EZH2, and SNON. (A) SMAD2/3, OCT4, NANOG, SOX2 and EZH2 bind to CDKI loci in human pluripotent cells. Histone H3K4me3 and H3K27me3 mark CDKI loci in pluripotent cells. SNON data represents leukemia cells. (B and C) OCT4 and NANOG form a complex with SMAD2/3 on CDKI loci in hPSCs. Sequential ChIP of (B) OCT4 and SMAD2/3 or (C) NANOG and SMAD2/3 in hPSC was performed and analyzed by Q-PCR. Significant differences compared to IgG/SMAD2/3 sequential ChIP sample calculated by *t*-test are marked. (D) OCT4 knockdown results in reduced SMAD2/3, EZH2, and SNON binding on CDKI loci. ChIP of OCT4, SMAD2/3, and SNON was performed in Scramble and OCT4 KD cells to test their presence on the regions uncovered by genome-wide SMAD2/3 and OCT4 ChIP-seq experiments. Significant differences calculated by *t*-test are marked. ns, not significant. (E) Immunostaining of p18 protein expression. Scale bar, 100 μm. (F) OCT4 knockdown causes an increase in CDKIs except for p27 in hPSCs. Scramble and OCT4 KD cells were analyzed by Q-PCR to determine the expression of CDKIs. (G) OCT4 knockdown causes an increase in CDKIs except for p27 in hPSCs. Scramble and OCT4 KD cells were analyzed by Q-PCR to determine the expression of CDKIs. Significant differences calculated by *t*-test are marked. ns, not significant. (H) OCT4 knockdown causes a decrease in repressive bivalent mark H3K27me3 on *p15*, *p18*, *p21,* and *p57* loci. Scramble and NANOG KD cells were analyzed by ChIP-QPCR of H3K4me3 and H3K27me3 marks on CDKI loci. Significant differences calculated by two-way ANOVA are marked. (I) OCT4 and NANOG represses *p15*, *p18,* and *p57* promoters. hPSCs were cotransfected with CDKI promoter-luciferase constructs and NANOG and/or SNON overexpression constructs. Luciferase signals were analysed 48 h after transfection. Experiments represent three replicates. Statistical analysis was performed by two-way ANOVA with multiple comparisons with Tukey correction and **** marks adjusted *P*-value < 0.0001, *** is adjusted *P*-value < 0.001, ** is adjusted *P*-value < 0.01, * is adjusted *P*-value < 0.05.

To validate the interconnection of ACTIVIN/TGFβ/NODAL-SMAD2/3 signaling and CDKIs, we analyzed SMAD2/3 ChIP-seq data in hPSCs. Interestingly, we found SMAD2/3 binding onto the regulatory regions in the proximity of p14/p16, p15, p18, p21, p27, and p57 loci in pluripotent cells despite the absence of active CDKI expression in pluripotent condition (Fig. 3A). SMAD2/3 ChIP-QPCR every 12 h after induction of endoderm specification showed binding already in self-renewing pluripotent hPSCs but also during endoderm differentiation (Fig. S3K). Promoter-luciferase assays further indicated the existence of an ACTIVIN/TGFβ/NODAL signaling dependent and SMAD2/3-mediated induction of CDKIs through their promoter regions in endoderm cells but not in hPSCs for p15, p18, and p57 (Fig. S3L and S3M), suggesting that ACTIVIN/NODAL-SMAD2/3 signaling could directly control the expression of CDKIs differently in pluripotent cells and differentiating cells.

Next, we also analyzed the binding of central transcription factors β-catenin, JUN, JUND, STAT3, and SMAD1 mediating the gene expression effects of differentiation signaling pathways of WNT, FGF2, PI3K, and BMP4 (Fig. S4A). Previously published ChIP-seq data analysis of β-catenin, JUN, JUND, STAT3, and SMAD1 in hESCs (GEO: GSM2945834) (Estaras et al., 2015; Pope et al., 2014; Tsankov et al., 2015), indicated the presence of binding peaks in the proximity of several CDKIs, and these binding peaks were extensively overlapping with each other, and with the binding of SMAD2/3 and EOMES. Therefore, we performed ChIP-qPCR of β-catenin, JUN, JUND, STAT3, and SMAD1 in undifferentiated hESCs and cells differentiated with the combined treatment of cytokines (Fig. S4B). ChIP-qPCR confirmed the binding of β-catenin, JUN, JUND, STAT3, and SMAD1 to CDKI loci indicating that these transcription factors mediate the gene expression changes of CDKIs during hPSC differentiation in cooperation with TGFβ-SMAD2/3.

Collectively, developmental signaling pathways, especially ACTIVIN/TGFβ-SMAD2/3, directly control the expression of CDKIs during stem-cell differentiation. However, it raised the intriguing question of the nature of the molecular mechanisms that provide stem cells with a distinct repressed CDKI expression signature that is rapidly altered upon the initiation of stem-cell differentiation.

### TGFβ/ACTIVIN-SMAD2/3-EZH2 cooperates with OCT4, NANOG, and SOX2 in self-renewing hPSCs to keep CDKIs in an epigenetically poised state for rapid activation

Since SMAD2/3 cooperates with sequence-specific transcription factors in gene regulation (Zwijsen et al., 1997), we decided to investigate this mechanism in CDKI regulation. By first performing transcription factor motif analyses within SMAD2/3 binding peaks we found the enrichment of OCT4, NANOG, and SOX2 binding motifs at the SMAD2/3 binding peaks at CDKI regulatory regions, suggesting that the core pluripotency transcription factors OCT4, NANOG and SOX2 could cooperate with SMAD2/3 in controlling CDKI expression in self-renewing pluripotent stem cells. SMAD2/3 is integrated into the “stemness” network in hPSCs and is involved in maintaining pluripotency by co-occupying loci that support self-renewal and the pluripotent state together with the key pluripotency factor NANOG, while at the same time allowing developmental genes to be rapidly induced upon differentiation (Brown et al., 2011; Pauklin and Vallier, 2015; Vallier et al., 2009a). Hence, CDKIs could represent genes that are similarly regulated by these stemness pathways.

ChIP-sequencing analysis of OCT4, NANOG, and SOX2 revealed their binding on most CDKI loci in hPSCs to overlapping regions with SMAD2/3 (Fig. 3A). Since SMAD2/3, NANOG, and OCT4 all bind to CDKI regulatory regions, we decided to explore the possible cooperation between SMAD2/3 and NANOG on CDKI loci in hPSCs. Sequential ChIP of OCT4 followed by SMAD2/3 or vice versa (Figs. 3B and S4C) and NANOG ChIP followed by SMAD2/3 or vice versa (Figs. 3C and S4D) showed that these stemness factors form a transcriptional complex on CDKI loci *p18*, *p15*, *p27*, and *p57*.

Next, to determine the effect of the core pluripotency factors on CDKI expression, we focused on OCT4 and NANOG, and performed its knockdown in hPSCs. SMAD2/3 and OCT4 CHIP experiments in Scramble and OCT4 iKD cells indicated reduced SMAD2/3 binding to CDKI loci in the absence of OCT4 (Fig. 3D). CDKI loci are also marked by both activating H3K4me3 and repressing EZH2-dependent H3K27me3 modifications (Fig. 3A), thus representing bivalent marks that are usually associated with developmental loci that are induced during tissue specification (Blanco et al., 2020). OCT4 iKD reduced the binding of EZH2 on CDKI loci (Fig. 3D), suggesting its involvement in regulating the deposition of repressive histone mark H3K27me3 on CDKI loci. Next, we investigated the effect of OCT4 knockdown on CDKI expression. OCT4 iKD led to the upregulation of CDKIs both at the mRNA and protein levels (Fig. 3E and 3F). We also observed a decrease in the EZH2-regulated repressive H3K27me3 bivalency mark on *p18*, *p15*, *p21,* and *p57* loci whereas the activating H3K4me3 bivalency mark showed no changes (Fig. 3H). Co-transfection of an OCT4 expression vector and *p18*, *p15,* and *p57* promoter-luciferase constructs reduced luciferase signal upon transient overexpression of OCT4 (Fig. 3I).

SMAD2/3 and NANOG CHIP experiments also indicated reduced SMAD2/3 binding to CDKI loci in the absence of NANOG (Fig. S4E). Furthermore, NANOG KD led to the upregulation of CDKIs both at the mRNA and protein levels (Fig. 3G), and decreased in the repressive H3K27me3 bivalency mark on *p18*, *p15*, *p21,* and *p57* loci whereas the activating H3K4me3 bivalency mark showed no changes (Fig. S4F). As for OCT4, co-transfection of a NANOG expression vector and *p18*, *p15,* and *p57* promoter-luciferase constructs showed a reduced luciferase signal upon transient overexpression of NANOG (Fig. 3I).

Altogether, these data uncovered that the SMAD2/3-OCT4-NANOG-EZH2 complex seems to maintain the characteristic CDKI expression pattern in hPSCs. However, the precise epigenetic mechanisms mediating the changes in CDKI expression still remained unclear.

### SMAD2/3-EZH2 maintains bivalent chromatin signatures on CDKI loci in self-renewing stem cells together with SNON

The SMAD2/3 repressor protein SNON/SKIL has been shown to suppress primitive streak and definitive endoderm genes in hPSCs, which helps to maintain pluripotency by suppressing differentiation (Tsuneyoshi et al., 2012). We, therefore, hypothesized that SNON could also mediate the transcriptionally suppressive function of SMAD2/3-NANOG-OCT4 complex on CDKI loci. SNON ChIP data analyses in leukemic cells revealed its potential binding to the same regions as SMAD2/3, OCT4, and NANOG (Fig. 3A), although no such information was available for hPSCs. Hence, we performed SNON ChIP qPCR experiments that indicated its enrichment on *p15*, *p18*, *p21* and *p57* loci, which was lost upon OCT4 KD (Fig. 3D) and NANOG KD (Fig. S4E), and ACTIVIN/TGFβ-SMAD2/3 pathway inhibition with SB431542 (Fig. S4H), indicating that NANOG and SMAD2/3 are involved in the recruitment of SNON onto CDKI loci. Moreover, co-transfection of CDKI promoter-luciferase constructs with an SNON expressing plasmid reduced the luciferase signal (Fig. 3I), indicating its repressive function on CDKI loci in hPSCs.

Next, we investigated the dependency of EZH2 on ACTIVIN/TGFβ-SMAD2/3 pathway. EZH2 showed binding to CDKI loci in hPSCs but this binding was reduced by SB431542 (Fig. S4I). Similarly, SB431542 treatment reduced the abundance of EZH2-dependent repressive histone mark H3K27me3 on CDKI loci in hPSCs (Fig. S4J), showing that TGFβ/Activin-SMAD2/3 signaling regulates the binding of EZH2 and H3K27me3 abundance on CDKI loci. To gain insight into the repressive effects of EZH2 and SNON and if they can be decoupled from each other, we used SNON knockdown cells and treated these with EZH2 chemical inhibitors, followed by the analysis of CDKI expression (Fig. S4K) and endoderm marker expression (Fig. S4L). The combination of SNON KD with EZH2 inhibitor GSK343 treatment had a stronger positive effect on p15, p18, and p57 induction compared to SNON KD alone or EZH2 inhibitor treatment. Furthermore, increased induction of endoderm markers T, EOMES, and SOX17 were observed upon SNON KD together with EZH2 inhibition than for either treatment alone. These data suggest that the repressive function of EZH2 and SNON can be decoupled and in CDKI and endoderm marker regulation, they provide additive repressive effects. Both SNON KD and EZH2 inhibition also led to the reduction of bivalent H3K4me3 and H3K27me3 marks on CDKI loci analyzed by sequential H3K4me3/H3K27me3 ChIP (Fig. S4M). Sequential ChIP between SMAD2/3 and EZH2 in hPSCs both ways (Fig. S5A and S5B), and sequential ChIP between SMAD2/3 and SMYD2 in endoderm cells both ways (Fig. S5C and S5D) further confirmed that SMAD2/3 forms a protein complex with EZH2 in hPSCs, and a complex with SMYD2 in endoderm cells. Thus, SNON and EZH2 support the pluripotent state by restricting the induction of CDKIs in hPSCs via a transcriptionally repressive complex together with SMAD2/3 and the core pluripotency factors.

Collectively, our results uncover an intricate control of CDKI expression in hPSCs: the expression of other CDKIs is maintained in a poised state in pluripotent cells by an OCT4-NANOG-SMAD2/3-EZH2-SNON complex.

### SMAD2/3 switches its binding to SMYD2 and EOMES to induce CDKI expression during the initiation of endoderm differentiation

ACTIVIN/TGFβ/NODAL has seemingly opposing functions in maintaining hPSCs in their pluripotent state but also inducing their differentiation to endoderm. TGFβ/ACTIVIN-SMAD2/3 signaling is crucial for regulating the self-renewal and pluripotency of hPSCs by SMAD2/3-mediated transcriptional regulation of stem-cell loci (Bertero et al., 2015), whereas it also induces definitive endoderm formation and EMT thus leading to the formation of endoderm cells with mesenchymal characteristics (Madrigal et al., 2023). This raises the question of how CDKI expression is regulated in pluripotent versus differentiating cells.

**Figure 4.**
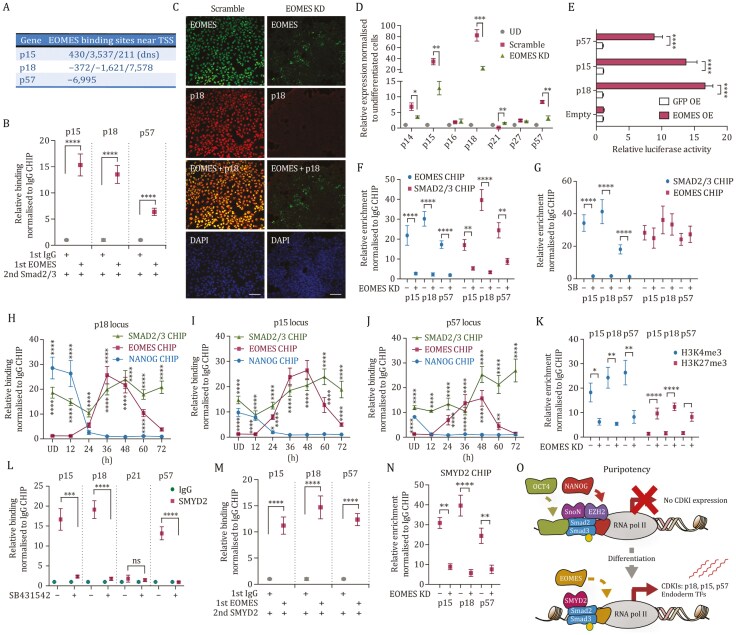
CDKIs are induced during endoderm differentiation by SMYD2-SMAD2/3-EOMES transcriptional complex. (A) The location of EOMES binding sites in the proximity of CDKI loci relative to their transcription start site. Putative EOMES binding sites were uncovered by genome-wide analysis of EOMES binding in Day 2 endoderm cells. (B) EOMES and SMAD2/3 form a complex on CDKI loci in endoderm cells. Sequential ChIP of EOMES and SMAD2/3 in Day 2 endoderm was followed by Q-PCR analysis. Significant differences compared to IgG/SMAD2/3 sequential ChIP sample and calculated by *t*-test are marked. (C) EOMES knockdown in endoderm causes the loss of p18 expression. Immunostaining of p18 and EOMES in Scramble and EOMES KD cells differentiated to endoderm for 2 days. Scale bar, 20 μm. (D) EOMES knockdown causes the loss of CDKI expression in endoderm cells. EOMES KD and Scramble cells were differentiated to endoderm and analyzed by Q-PCR for determining CDKI expression. Significant differences compared to the Scramble shRNA sample and calculated by *t*-test are marked. (E) EOMES induces CDKI expression via binding to regulatory regions on CDKI loci. CDKI promoter-luciferase construct harboring putative EOMES binding regions were co-transfected with EOMES-expressing vector or GFP into Day 2 endoderm cells and analyzed 24 h after transfection. Significant differences compared to the OE GFP sample and calculated by *t*-test are marked. (F) SMAD2/3 binding to *p15*, *p18,* and *p57* loci in endoderm depends on EOMES. SMAD2/3 and EOMES CHIP were performed in Scramble and EOMES KD cells in Day 2 endoderm cells and analyzed by Q-PCR. Significant differences compared to the Scramble shRNA sample and calculated by *t*-test are marked. (G) Blocking SMAD2/3 binding to CDKI loci by inhibiting ACTIVIN/NODAL signaling with SB431542 (SB) does not abolish EOMES binding. Day 2 endoderm cells were treated with SB431542 for 2 h and analyzed by SMAD2/3 ChIP or EOMES ChIP. Significant differences compared to not SB431542 treated endoderm cells and calculated by *t*-test are marked. (H–J) Timeline of SMAD2/3, NANOG, and EOMES binding to *p18*, *p15,* and *p57* loci during endoderm differentiation. (K) EOMES knockdown in endoderm cells results in the loss of activating histone mark H3K4me3 on CDKI loci and elevation of the repressing histone modification H3K27me3. Scramble and EOMES KD cells were differentiated to Day 2 endoderm and analyzed by H3K4me3 ChIP and H3K27me3 ChIP followed by Q-PCR. Significant differences compared to the Scramble shRNA sample and calculated by *t*-test are marked. (L) SMYD2 binding to *p15*, *p18,* and *p57* loci is reduced by ACTIVIN/NODAL signaling inhibitor SB431542. (M) EOMES and SMYD2 form a complex on CDKI loci in endoderm cells. Sequential ChIP of EOMES and SMYD2 in Day 2 endoderm was followed by Q-PCR analysis. Significant differences compared to IgG/SMYD2 sequential ChIP sample and calculated by *t*-test are marked. (N) SMYD2 binding to CDKI loci is reduced upon EOMES KD in endoderm. Significant differences compared to Scramble shRNA sample and calculated by *t*-test are marked. (O) Graphical model depicting the absence of CKI expression in pluripotent cells and induction in endoderm cells by SMAD2/3 and EOMES. During endoderm differentiation, EOMES-SMAD2/3 complex binds to *p18*, *p15,* and *p57* loci and induces their expression along with the endoderm genes. All data are shown as mean ± s.d. (*n* = 3).

SMAD2/3 ChIP-seq analyses revealed the binding of SMAD2/3 to the CDKI regulatory regions in endoderm cells, some of which were overlapping with the binding regions found in pluripotent cells whereas there were also some new binding sites (Fig. 3A). The motif analyses of SMAD2/3 peaks in the proximity of CDKI loci in endoderm indicated the presence of transcription factor EOMES with locations close to the CDKI Transcription Start Sites (TSS) (Fig. 4A). Since EOMES is not expressed in hESCs, this supported the notion that SMAD2/3 can switch its transcriptional partners during differentiation and this mechanism is essential for endoderm specification (Brown et al., 2011; Madrigal et al., 2023; Mullen et al., 2011; Teo et al., 2011). Indeed, we have shown previously that SMAD2/3 interacts with NANOG in hPSCs to maintain pluripotency but with EOMES to drive endoderm formation (Madrigal et al., 2023; Teo et al., 2011). Thus, we hypothesized that SMAD2/3 could cooperate with EOMES to induce CDKI expression upon differentiation. EOMES ChIP-Seq analyses in endoderm showed that this master regulator of differentiation can indeed be found on *p18*, *p15,* and *p57* loci on regions overlapping with SMAD2/3 binding, and ChIP-QPCR validated these observations (Fig. 3A). Importantly, sequential ChIP of EOMES and SMAD2/3 demonstrated that these transcription factors are part of the same protein complexes co-binding these genomic regions (Fig. 4B). Furthermore, knockdown of EOMES expression during endoderm differentiation resulted in a decrease of CDKI expression (Figs. 4C, 4D and S5E). Promoter-luciferase assays provided further evidence that EOMES is able to directly induce p15, p18, and p57 expression in endoderm cells (Fig. 4E).

To determine the molecular function of EOMES in the induction of CDKI expression, we performed SMAD2/3 ChIP experiments in hPSCs knocked down for EOMES and differentiating toward the endoderm lineage. These experiments revealed that EOMES is necessary for the recruitment of SMAD2/3 to these loci (Fig. 4F). Reversely, inhibition of SMAD2/3 did not affect the binding of EOMES (Fig. 4H). Thus, EOMES seems to be necessary for SMAD2/3 binding on CDKI loci but not vice versa. To characterize the timing of NANOG and EOMES on CDKI loci, we performed ChIP-QPCR of these factors every 12 h during endoderm differentiation (Fig. 4H–J). NANOG binding to CDKI loci was lost by 24 h of endoderm differentiation while EOMES binding started to increase at this time point on CDKI loci, indicating an exchange of factors from the key pluripotency factor NANOG to the inducer of definitive endoderm EOMES. At the same time, SMAD2/3 binding was detected in pluripotent conditions as well as during endoderm differentiation (Fig. 4H–J).

Next, we analyzed the deposition of histone modification on CDKI loci during endoderm differentiation and the effect of EOMES. The knockdown of EOMES led to the reduction of transcriptionally activating modification H3K4me3 whereas there was a concomitant increase in repressive H3K27me3 (Fig. 4K). This led us to investigate the binding of SMYD2 on CDKI loci since we had identified it in compound screening and showed its function as a regulator of CDKI loci. ChIP-qPCR indicated that SMYD2 bound to the same regions near CDKI loci as SMAD2/3 and EOMES, and the inhibition of ACTIVIN/TGFβ signaling with SB431542 reduced its binding. This suggested that SMAD2/3 helps to recruit SMYD2 to CDKI regulatory regions (Fig. 4L). In addition, sequential ChIP of EOMES and SMYD2 demonstrated that these factors are part of the same protein complexes co-binding CDKI genomic regions (Fig. 4M). Lastly, SMYD2 binding is decreased to CDKI loci upon EOMES KD, indicating that EOMES contributes to SMYD2 recruitment to CDKI loci during endoderm differentiation.

Collectively, these data suggest that pluripotency factors such as NANOG with EZH2 and SNON are replaced on CDKI loci during the switching from self-renewal to differentiation, whereas EOMES together with SMAD2/3 and SMYD2 cooperate to directly induce the expression of p15, p18, and p57 CDKI during endoderm differentiation (Fig. 4O).

### CDKIs direct lineage specification of human pluripotent stem cells

**Figure 5.**
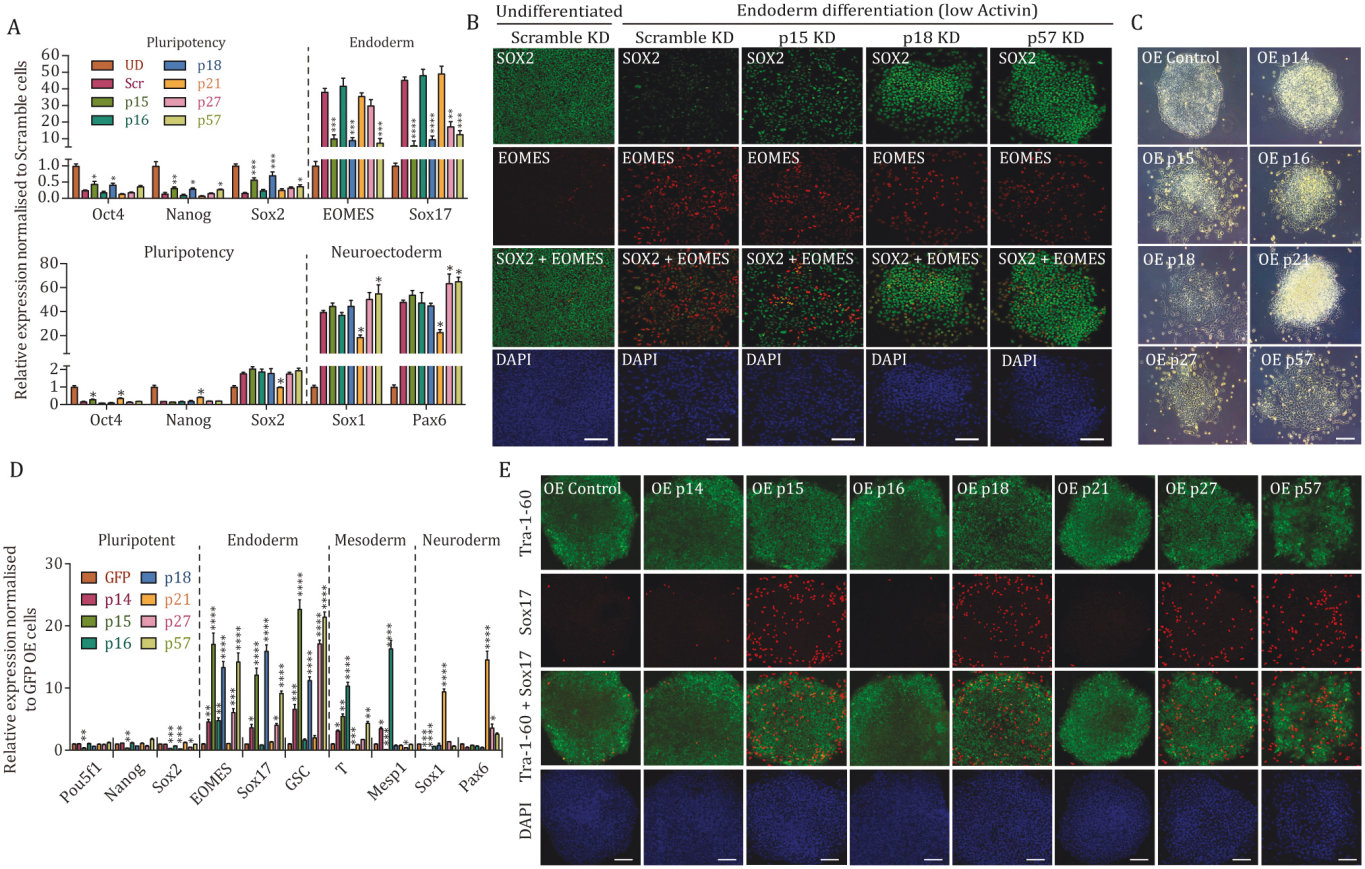
CDKI expression affects the differentiation efficiency toward distinct cell fates. (A) p15, p18, and p57 knockdown reduces the efficiency of endoderm differentiation while p21 knockdown reduces the efficiency of neuroectoderm differentiation. Q-PCR analysis of pluripotency genes and germ layer-specific markers in CDKI KD cells differentiated toward endoderm and neuroectoderm. Significant differences compared to differentiated Scramble shRNA samples calculated by *t*-test are marked. (B) p15, p18, and p57 knockdown cells have decreased endoderm specification capacity. Immunostaining of pluripotency and differentiation markers in Scramble and CDKI KD cells. Scale bar, 100 μm. (C) Overexpression of CDKI protein causes a loss of pluripotency and elevated differentiation morphology of hESCs as shown in representative colonies of OE CDKIs. (D and E) Overexpression of p15, p18, and p57 induces endoderm differentiation. Differentiation markers were analyzed by (D) mRNA expression of pluripotency and differentiation markers in OE GFP and OE CDKI cells or (E) immunostaining. Scale bar, 100 μm. Significant differences compared to OE GFP calculated by *t*-test are marked.

The germ layer-specific induction of CDKIs during hPSC differentiation suggested a potential function for them not only in cell cycle regulation but also in cell-fate decisions. Therefore, we decided to determine the role of CDKIs during endoderm differentiation using gain- and loss-of-function studies. We first knocked down each individual CDKI (p14/p16, p15, p18, p21, p27, and p57; Fig. 5A) using stable overexpression of shRNA as described previously (Pauklin and Vallier, 2013). A decrease in expression (>80%) in Day 2 differentiated cells was confirmed for CDKIs at both protein and mRNA levels (Fig. S6A and S6B). As expected due to the absence of CDKI expression in hPSCs, the morphology and number of pluripotent colonies derived after transfections were not affected when compared to control, except for a decrease in p27 (Fig. S6C and S6D), suggesting that p27 could impact hPSCs self-renewal. Nonetheless, Q-PCR analyses for assessing the background differentiation revealed that hPSCs with a knockdown for the expression of p15, p18, and p57 show a decrease in endoderm marker expression (SOX17, EOMES, and GSC; Fig. S6E) while neuroectoderm markers PAX6 and SOX1 were increased (Fig. S6E). On the other hand, hPSCs with a knockdown for p21 expression showed the opposite effect, and p16 knockdown was associated with a decrease in mesoderm markers such as Brachyury/T and Mesp1 (Fig. S6E). Finally, knockdown of p27 only caused a limited change in differentiation marker expression (Fig. S6E). Flow cytometry analyses confirmed these observations (Fig. S6F–I) thereby demonstrating that a decrease in CDKI family members increased specific background of differentiation.

To determine the function of CDKIs on germ layer specification, hPSCs with a knockdown for p14/p16, p15, p18, p21, p27, and p57 expression were differentiated into endoderm, mesoderm, and neuroectoderm. Q-PCR analyses and flow cytometry revealed that the absence of p18, p15, and p57 reduced the capacity of hPSCs to differentiate into endoderm and into mesoderm as shown by a decrease in mesendoderm markers and an increase in pluripotency markers (Figs. 5A, S7A and S7B). On the other hand, the same hPSC lines displayed an enhanced capacity to differentiate toward the neuroectoderm lineage (Fig. 5A). In contrast, loss of p21 improved endoderm differentiation of hPSCs while neuroectoderm specification was less efficient and mesendoderm was not affected (Figs. 5A, S7A and S7B). Finally, p16 KD improved mesoderm specification while other germ layers were not significantly affected (Figs. 5A, S7A and S7B). We confirmed these results at the protein level by flow cytometry (Fig. S7D–G) and immunostaining (Fig. 5B). Finally, we performed teratoma assays in mice on hPSCs knocked down for p18, p21, and p57 expression (Fig. S7H and S7I). Histological analyses revealed that the tumors generated from hPSC with a knockdown for p18 and p57 contain an increased quantity of neuroectodermal tissues to the detriment of endodermal tissues. In contrast, hPSCs with a knockdown for p21 produced more mesoderm and endoderm derivatives and less neuroectoderm when compared to teratomas from Scramble shRNA. Considered together, these data demonstrate that CDKIs impact germ layer differentiation *in vitro* and that a specific combination of CDKIs is involved in hPSC differentiation to achieve particular cell-fate choice.

To further confirm this hypothesis, we investigated the effects of CDKI overexpression on differentiation of hPSCs. For that, we expressed individual CDKIs constitutively in hPSCs (Fig. S8A) as previously described (Pauklin and Vallier, 2013). CDKI overexpression strongly reduced colony formation after transfection (p21, p27, p57, p18, and p15 in Fig. S8B) suggesting a negative effect on cell survival and/or self-renewal. The remaining colonies displayed a level of CDKIs comparable or lower than the natural level of expression observed in endoderm cells with the exception of p14 that had higher expression (Fig. S8C). As expected, CDKI overexpressing hPSCs grew very slowly and overexpression of p15, p18, and p57 resulted in the lengthening of the G1 phase while other CDKIs had weaker effects (Fig. S8D and S8E). CDKI overexpressing hPSCs were also strikingly more prone to spontaneous differentiation (Fig. 5C). These observations were confirmed by immunostaining and Q-PCR analyses showing that overexpression of p15, p18, and p57 increased the background expression of endoderm markers while reducing pluripotency and neuroectoderm markers (Fig. 5D and 5E). p16 overexpression modestly induced mesoderm markers while blocking neuroectoderm. Only p21 overexpression induced the neuroectoderm markers PAX6. These results were further confirmed by flow cytometry analyses (Fig. S8F–I).

Next, we studied the effect of CDKI overexpression during hPSC specification toward endoderm, mesoderm, and neuroectoderm. Flow cytometry analyses and immunostaining showed that overexpression of p15, p18, and p57 during endoderm differentiation with a less than optimal concentration of Activin A, which results in inefficient differentiation, increased endoderm marker expression while decreasing pluripotency markers (Fig. S9A–I). Furthermore, the same overexpression decreased neuroectoderm differentiation efficiency while increasing pluripotency markers (Fig. S9A–I).

Collectively, these functional studies showed that CDKIs promote or limit the specification of hPSCs toward specific germ layers while determining the length of their cell cycle.

### CDKI-mediated inhibition of CDK4/6 increases SMAD2/3 transcriptional activity

**Figure 6.**
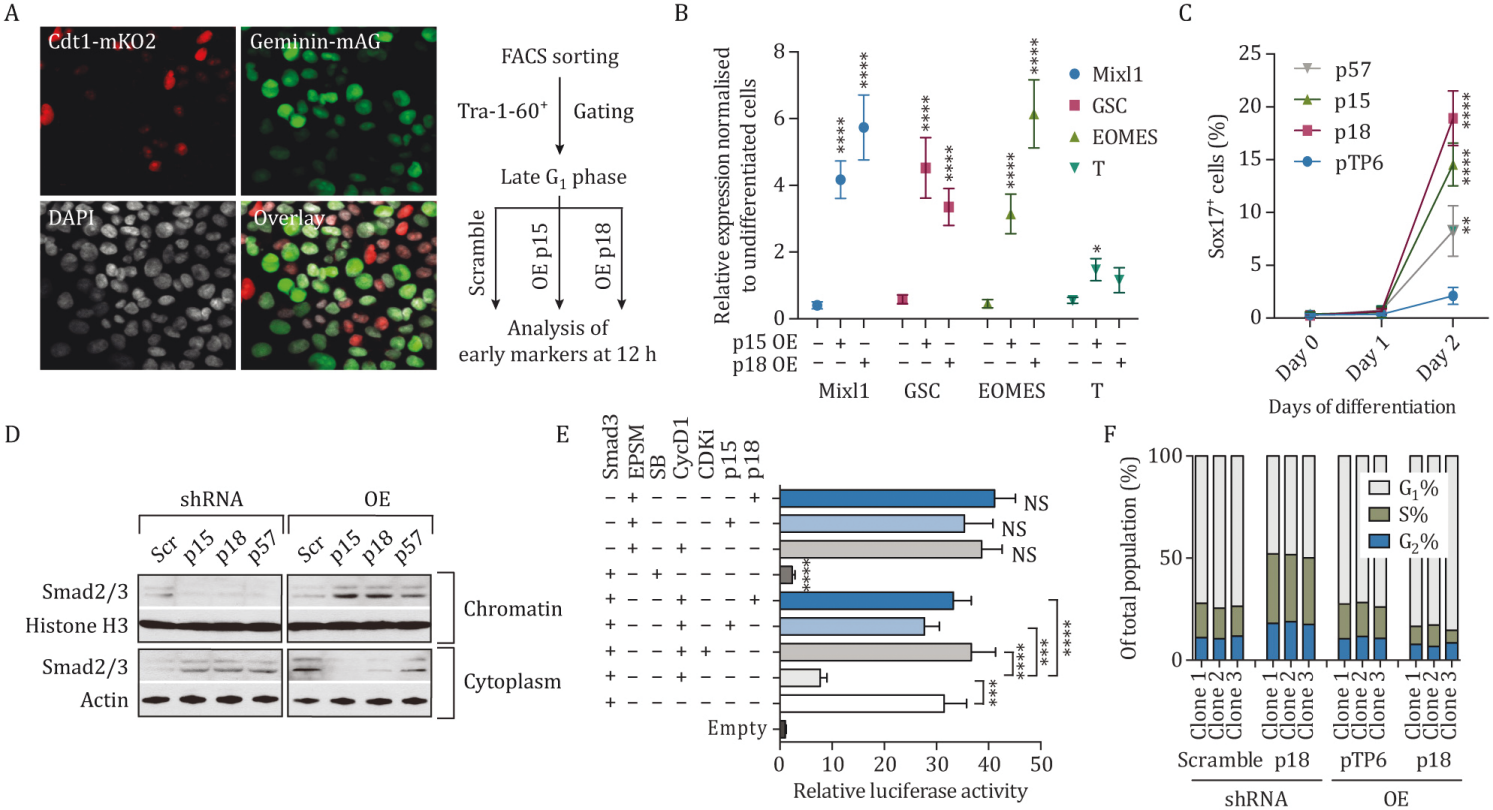
Positive feedback loops between SMAD2/3-CDKI and developmental transcription factors. (A) Analysis of cell-cycle dependent differentiation potential in FUCCI-hPSCs. Cells were transfected with CDKI expression constructs and synchronized by cell sorting to early G_1_ phase 48 h after transfection. Thereafter, cells were differentiated into endoderm and analyzed for endoderm markers. Scale bar, 20 μm. (B) CDKI expression in late G_1_ allows for the induction of endoderm differentiation. Q-PCR analysis of early endoderm marker induction in control cells, p18 OE and p15 OE cells. Significant differences compared to OE vector calculated by *t*-test are marked. (C) CDKI expression in late G_1_ phase induces endoderm differentiation. Cells were treated as in (A) and analyzed for SOX17 expression by flow cytometry at 24 h and 48 h time points. Significant differences compared to OE pTP6 vector calculated by two-way ANOVA are marked. (D) Relative abundance of SMAD2/3 in the cytoplasm and on chromatin upon CDKI knockdown or overexpression. Histone H3 and ACTIN are used as loading controls. (E) CDKIs induce SMAD2/3 transcriptional activity via inhibiting Cyclin D-CDK4/6 mediated phosphorylation of SMAD2/3 in its linker region. Cells were co-transfected with CDKI expressing plasmids and a luciferase construct under the regulation of a consensus SMAD binding element. Significant differences calculated by *t*-test are marked. (F) p18 expression regulates G_1_ phase length during endoderm differentiation. Cell-cycle profile of p18 KD and p18 OE cells was compared to control cells by EdU incorporation.

We decided to delineate further the molecular mechanisms by which CDKIs could influence cell-fate choice of hPSCs. We have shown previously that CDK4/6-Cyclin D complex can regulate the transcriptional activity associated with ACTIVIN/NODAL signaling by inhibiting the cytoplasmic-nuclear shuttling of SMAD2/3 (Pauklin and Vallier, 2013) during the late part of the G_1_ phase. Therefore, we hypothesized that CDKIs could influence hPSC fate choice by blocking Cyclin D-CDK4/6 inhibition of SMAD2/3. To validate this hypothesis, we transitorily overexpressed p18 or p15 in FUCCI-hPSCs and then studied the capacity of the resulting cells to initiate endoderm differentiation during the late G_1_ phase when Cyclin D-CDK4/6 block SMAD2/3 activity (Fig. 6A–C). Transient expression of CDKIs in the late G_1_ phase enhanced endoderm marker expression as analyzed by Q-PCR and flow cytometry. Furthermore, SMAD2/3 ChIP-QPCR in the early and late G_1_ phase revealed that p18 overexpression allows SMAD2/3 binding to the endoderm loci EOMES, Mixl1, and GSC (Fig. S10A) while SMAD2/3 is usually blocked to access these genomic regions by cyclin D-CDK4/6 activity (Pauklin and Vallier, 2013). We also performed SMAD2/3 ChIP-QPCR at different time points during endoderm differentiation of hPSCs with a knockdown for p18, p15, and p57, and observed that SMAD2/3 binding to endodermal loci MIXL1, EOMES, and SOX17 was strongly reduced (Fig. S10B–D). Next, we determined the subcellular localization of SMAD2/3 during differentiation in the presence of absence of CDKIs. These experiments showed that knockdown of CDKI expression caused cytoplasmic accumulation of SMAD2/3 away from chromatin during endoderm differentiation, while overexpression of the same CDKIs had the reverse effect, resulting in SMAD2/3 accumulation onto chromatin (Figs. 6D and  S10E). Furthermore, overexpression of p15, p18 and p57 increased the transcriptional activity of SMAD3 (Figs. 6E and  S10F) while CDKIs did not affect the transcriptional activity of SMAD3-EPSM which contains mutations at CDK4/6 phosphorylation sites (T178V, S203A, S207A, S212A; (Kretzschmar et al., 1999)) (Fig. 6E), suggesting that these linker residues mediate CDKI effects on SMAD2/3. These results demonstrate that the CDKIs promote the activity of Activin/Nodal signaling by limiting the inhibitory action of Cyclin D-CDK4/6 on SMAD2/3-mediated transcription.

Importantly, the induction of CDKIs upon differentiation also results in the lengthening of the G_1_ phase. Cell-cycle analyses showed that overexpression of p18 lengthened the G_1_ phase of differentiating endoderm cells while the knockdown of the same CDKI decreased the size of G_1_ (Fig. 6F). This change in the cell-cycle profile could enable SMAD2/3 to bind for a prolonged period of time onto endodermal genes and thus to increase the expression of master regulators necessary for cell-fate commitment. Following this hypothesis, we explored if endoderm-promoting CDKIs (p15, p18, and p57) could affect SMAD2/3 binding and activity during germ layer specification. Since endoderm differentiation is accompanied by a significant extension of the G_1_ phase length, we wanted to gain insight into the dynamics of SMAD2/3 activity according to the cell cycle phases. For this, we transfected FUCCI-hPSCs with the SBE4-luciferase construct and analyzed SMAD2/3 transcriptional activity in pluripotent cells and Day 1 endoderm in the presence or absence of p18 overexpression (Fig. S11A). Luciferase assay revealed that the transcriptional activity of SMAD2/3 is elevated in endoderm cells overexpressing p18 and thus displaying an increased G_1_-enhanced SMAD2/3 activity. Next, we analyzed the dynamics of SMAD2/3 binding on endoderm loci during differentiation in the presence or absence of p18 overexpression (Fig. S11B). SMAD2/3 ChIP-QPCR revealed a transient binding of SMAD2/3 to its developmental target loci in the early G_1_ phase of pluripotent cells, while its binding was extended across the whole G_1_ phase in endoderm cells (Fig. S11B), especially in the presence of p18. Similar experiments performed on H3K36me3 revealed that an increase in this histone modification marking active transcription was not only concomitant with elevated SMAD2/3 binding in the extended G_1_ phase of endoderm cells but was also further enhanced by p18 overexpression. Importantly, similar observations were made with the genomic regions including the cell cycle inhibitor *p15*, *p18,* and *p57* loci (Fig. S11C), showing that SMAD2/3 activity is increased by CDKIs and in turn, SMAD2/3 induces CDKI expression thereby forming a regulatory circuitry with these cell-cycle inhibitors (Fig. S11C). Altogether, these results show that CDKIs limit the activity of Cyclin D/CDK4-6 complexes, which concomitantly increase the length of the G_1_ phase both of which increase of transcriptional activity of SMAD2/3 (Fig. S11D).

### Manipulation of EZH2, SMYD2, and SMAD2/3-CDKI can be harnessed for guiding tissue self-formation for biomedical applications

**Figure 7.**
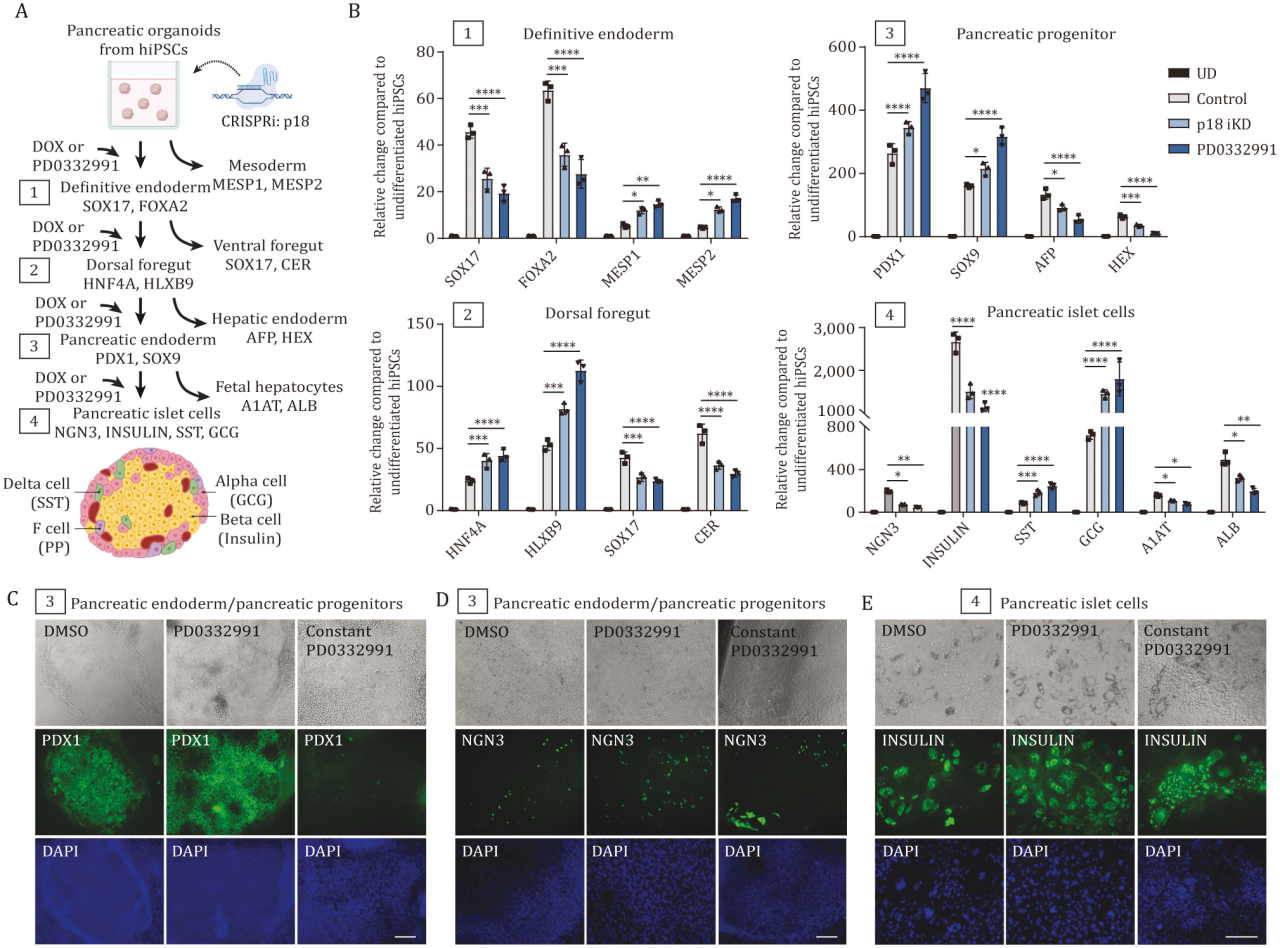
Manipulating EZH2, SMYD2, and SMAD2/3-CDKI activity can be harnessed for guiding tissue self-formation for biomedical applications. (A) Schematics of hIPSC organoid differentiation toward pancreatic tissue. Each DOX or PD0332991 treatment during the 1–4 specification stages lasted for 72 h. Markers for each cell identity are shown. (B) QPCR analyses of markers during the pancreatic differentiation and the alternative cell-fate markers were analyzed at 1–4 specification stages. The treatments for DOX-mediated p18 knockdown and CDK4/6 inhibition with PD0332991 are shown in (B). (C–E) Dynamic PD0332991 treatment improves pancreatic differentiation while constant PD0332991 treatment impairs differentiation as evidenced by reduced expression and altered spatial distribution of key pancreatic markers PDX1, NGN3, and INSULIN. Experiments represent three replicates. Statistical analysis was performed by two-way ANOVA with multiple comparisons with Tukey correction and **** marks adjusted *P*-value < 0.0001, *** is adjusted *P*-value < 0.001, ** is adjusted *P*-value < 0.01, * is adjusted *P*-value < 0.05.

Cells have a remarkable capacity to self-organize and form complex tissues without apparent external guidance, suggesting inherent developmental programs that are activated self-sufficiently in a step-wise manner. EZH2, SMYD2, and SMAD2/3-CDKI with developmental transcription factors could provide insight into the mechanisms of such developmental circuitries that can gradually drive cell-fate specification and complex tissue formation since each step initiates the next molecular process (Fig. S11E). This provides insight into developmental processes during organogenesis in early development. Hence, we hypothesized that it might be possible to make use of the discovered mechanisms for guiding tissue self-formation for biomedical applications. To begin to address this issue, we used an inducible p18 CRISPR/Cas-KRAB knockdown system that relies on the expression of p18 gRNAs under DOX-inducible conditions that can be turned on and off upon the addition of Doxycyclin. We established a p18 iKD in hIPSC line and also utilized the small molecule compound PD0332991 for inhibiting CDK4/6 by adding it to the media. We cultured the hIPSCs in organoid condition and adapted a previously published differentiation protocol (Madrigal et al., 2023; Pauklin and Vallier, 2013) for pancreatic insulin-producing beta-cells for this experimental condition (Fig. 7A). Each hiPSC step-wise differentiation stage, definitive endoderm, dorsal foregut, pancreatic progenitors/pancreatic endoderm, and pancreatic islet cells, were supplemented with either DOX for p18 knockdown, or with PD0332991 for CDK4/6 inhibition. QPCR analysis of these treatments indicated that p18 iKD and PD0332991 shift cell-fate specification by reducing specification toward pancreatic beta-cell fate, as in definitive endoderm (SOX17 and FOXA2) and pancreatic islet cell (NGN, INSULIN) specification stage. At the same time, p18 iKD and PD0332991 further shift cell-fate specification toward other possible cell specification routes (stage 1: mesoderm markers MESP1, MESP2; stage 4: alpha cell marker GSC and delta cell marker SST). In contrast, p18 iKD and PD0332991 treatment during dorsal foregut (HNF4A, HLXB9) and pancreatic progenitor (PDX1, SOX9) stage, improve cell-fate specification toward these cell identities while reducing the other possible cell-specification routes (stage 2: ventral foregut markers SOX17 and CER; stage 3: hepatic endoderm markers AFP and HEX). These results indicated that the dynamical activation and inactivation of the SMAD2/3-CDKI-developmental TF circuitry contributes to guiding cell-fate specification upon multiple routes available for the differentiating cells.

We examined the expression of some key markers by immunostaining and found that temporal CDK4/6 inhibition with PD0332991991 at pancreatic progenitor stage further improves the specification toward PDX1 positive pancreatic progenitor cells (Fig. 7C), NGN3 (Fig. 7D), and INSULIN expressing beta-cells at the islet cell specification stage (Fig. 7E). On the other hand, the constant inhibition of CDK4/6 with PD0332991 throughout the differentiation process reduces the formation of PDX1 positive cells, and interestingly, alters the spatial expression of NGN3 and INSULIN (Fig. 7D).

We also investigated the possible utility of modifying EZH2 or SMYD2 activity during pancreatic differentiation to islet cell type in hIPSCs by using small molecule inhibitors identified in our screening process. The treatment of cells at pancreatic differentiation stage 4 with SMYD2 inhibitors led to the reduction of insulin and NGN3 expression but increased expression of SST and GCG (Fig. S11F). This suggested that SMYD2 activity mediates the differentiation of Insulin-producing beta-cells of alpha and delta cells expressing SST and GCG, respectively. On the other hand, EZH2 inhibitors led to an increase in insulin, NGN3. SST and GCG, thus indicating that inhibiting EZH2 activity temporarily can improve the differentiation of hPSCs toward the cell types of pancreatic islets. Thus, SMYD2 and EZH2 small molecule compounds can be utilized to guide cell-fate specification and improve the production of cell types with biomedical interest.

Collectively, our results suggest that the dynamic fluctuation of SMYD2, EZH2, and CDK4/6 activity during pancreatic islet fate specification contributes to efficient differentiation. Furthermore, manipulation of the positive feedback loops between SMAD2/3-CDKI and developmental transcription factors can be intersected for guiding tissue self-formation for producing patient-specific cell types such as pancreatic islet cells that have biomedical applicability.

## Discussion

Our study has uncovered EZH2 and SMYD2 as novel epigenetic regulators of the switching from self-renewal to differentiation, and a function for cell-cycle inhibitors in directing lineage specification of human stem cells. Although our compound library had extensive coverage, the compound library still represents a subset of chromatin regulators as target factors that is missing certain chromatin regulators. This is due to the current state of research where not all chromatin regulators have functional small compounds available, and various inhibitors or functional antagonists are still in development. Therefore, we cannot rule out the roles of other major chromatin regulators, such as chromatin remodelers and mediator complexes that were not among the targets of the currently used functionally validated small compound library. Future research will aim to provide further insight to this aspect and fill in the gaps in knowledge.

Our results show that self-renewal and differentiation signals such as ACTIVIN maintain the stem-cell-specific expression profile of CDKIs. Accordingly, hPSCs keep most CDKIs in a poised state via NANOG-OCT4-SMAD2/3-EZH2-SNON complex and ready for rapid induction. In response to developmental signals that trigger stem-cell differentiation, the bivalently marked (H3K4me3 and H3K27me3) poised CDKIs are induced only few hours after the induction of differentiation via cooperation between SMAD2/3 and developmental master regulators such as EOMES and epigenetic regulator SMYD2. CDKIs can then inhibit in part the activity of the Cyclin D-CDK4/6 complex in the next G_1_ phase during which endoderm cells are specified. This mechanism allows CDKIs not only to extend the G_1_ phase but concomitantly enhance the transcriptional activity of ACTIVIN/NODAL/TGFß signaling which can then gradually activate the expression of master regulators of cell-fate choice such as SOX17. The transcriptional activity of SMAD2/3 proteins is linked to their shuttling from the cytoplasm to the nucleus where they bind to SMAD-binding elements at their target loci. Our data indicated elevated protein levels of SMAD2/3 in the nucleus following CDKI-mediated G_1_ lengthening since the EPSM mutants of SMAD2/3 showed high promoter-luciferase activity which was not impacted by p15 OE, p18 OE, Cyclin D1 OE, and CDK4/6 inhibition. Collectively, these data indicate that the heightened activity of SMAD2/3 upon CDKI-mediated G_1_ lengthening results from enhanced nuclear accumulation that are mediated by the EPSM residues phosphorylated by CDK4/6 on SMAD2/3 linker regions. This positive feedback loop appears to be important for hPSCs to commit toward the endodermal fate and to enable ACTIVIN/NODAL/TGFß signaling to convert from signaling that protects pluripotency to signaling that promotes differentiation (Fig. 7K). The switching of SMAD2/3 interaction partners from EZH2 to SMYD2 needs further investigation but we can hypothesize that it could be mediated by the changing expression of transcription factors and post-translational modification mediated by differentiation signaling pathways that crosstalk to each other. Upon differentiation, pluripotency factors such as SOX2, OCT4, and NANOG are rapidly repressed, and SKIL protein undergoes degradation. At the same time, differentiation signals lead to the induction of EOMES and other transcription facts. These changes in protein expression patterns could allow for the formation of new protein complexes together with post-translational modifications on SMAD2/3 that could potentially help to explain the switch of SMAD2/3 from EZH2 to SMYD2 during endoderm differentiation.

These mechanisms could explain how cell-fate commitment occurs upon differentiation of a diversity of stem cells. Indeed, TGFß signaling has been shown to directly control the expression of p15, p21, p27, and p57 in tissue-specific progenitors located in the central nervous system, bones, hematopoietic system, and liver (Chang et al., 2009; Palazuelos et al., 2014; Podkowa et al., 2013; Scandura et al., 2004). Therefore, the mechanisms uncovered by our current study could be relevant for a diversity of cell types in which the induction of CDKIs by TGFß will be not only important for blocking their proliferation but also for enhancing the activity of differentiation signals necessary for their final maturation/commitment.

Similarly, a decrease in CDKI expression is a common process in cancer and our results suggest that this mechanism could not only lead to increased capacity to proliferation but also a decreased capacity to sense differentiation signals. Indeed, TGFß signaling has been shown to control the expression of several CDKIs in a diversity of cancer lines (Scandura et al., 2004). As uncovered by our results, the maintenance of p27 expression in hPSCs via SMAD2/3-NANOG-OCT4 is particularly interesting considering the dual role of p27 as a tumor suppressor as well as an oncogene (Besson et al., 2007). It suggests that this molecular machinery could also play a role in promoting the proliferation of cancer cells or cancer stem cells. OCT4 and NANOG expression has indeed been linked to various tumors (Ibrahim et al., 2012; Jeter et al., 2009, 2011; Zhang et al., 2013). Furthermore, the suppression of other CDKIs by SMAD2/3-NANOG-OCT4-EZH2-SNON in hPSCs could also support the proliferation capacity of cancer stem cells, considering the tumor-promoting effects of SNON (Edmiston et al., 2005; He et al., 2003; Krakowski et al., 2005; Zhu et al., 2005). Hence, the results uncovered by our study shed light on the interconnection between cell-cycle regulation and cell-fate decisions, which are likely to be relevant for a broad range of cellular settings including stem cells, non-malignant progenitors, and cancer stem cells.

The role of CDKIs in embryogenesis is less clear since the absence of these proteins in mice does not result in severe phenotypes during early development. p18 is among the more abundantly expressed INK4-family CDKIs and is detected throughout mouse embryogenesis, p15 is less abundant, and p19(ARF)/p16 show relatively little expression in prenatal tissues while they become more abundantly expressed in adult tissue (Zindy et al., 1997) where they act as tumor suppressors (Kamijo et al., 1997; Serrano et al., 1996). Hence, among these CDKIs, p18 seems to play a more important role during early development, which is also underlined by the phenotype of the mouse knockout for p18, which shows an increased body size and widespread organomegaly (Franklin et al., 1998). Interestingly, p18 mRNA expression during development in the mouse matches our *in vitro* differentiation results for human stem cells in that it shows the strongest induction during differentiation among the CDKIs and is particularly expressed in the endoderm. However, it is known that CDKIs can have at least partially redundant activity, which could mask their function during gastrulation. For instance, mice with a knockout for p27 have an increased body size like p18 KO mice (Kamijo et al., 1997), while p18/p27 double-null mice have an even more increased body size compared to both single knockouts (Franklin et al., 1998). Such an additive effect on body size is not seen in p18/p21 double KO mice (Deng et al., 1995), suggesting CDKI-specific redundancy. Furthermore, p18 can cooperate with p27 during liver regeneration *in vivo* by regulating DNA synthesis and G_1_/S phase progression (Luedde et al., 2003), and it is likely to involve also p57 (Awad et al., 2000). Due to the emergence of CRISPR/Cas9-mediated genome-editing system (Hsu et al., 2014), the questions of CDKI cooperation in various tissues could be revisited by simultaneous multi-gene knockouts. These studies would also benefit from genome-wide analysis of epigenetic changes, which has become possible only in recent years (Rivera and Ren, 2013). While our study provides insight into the differentiation mechanism of hPSCs, it makes the *in vivo* relevance of our discoveries difficult to confirm. This could be partly overcome by using *in vivo* experiments in the mouse but there are potential species differences between human and other animal models which make a direct comparison challenging. Another limitation aspect relates to the applicability of our discoveries to other cellular differentiation stages such as later stages of development beyond the pancreatic–hepatic specification. Our current study does not provide evidence if our observations are more universally observed in organogenesis in other tissues, which could be investigated in future studies. Furthermore, the usage of small molecule compounds is potentially a useful practical strategy to guide cellular differentiation toward functional cell types with biomedical interest that goes beyond pancreatic islet cells such as insulin-producing beta-cells. Therefore, such studies could be expanded further in the future.

Importantly, our results also suggest that CDKI could function in a tissue-specific manner. Indeed, we observed that p21 was functionally important for neuronal differentiation while p16 promoted mesoderm and other CDKIs endoderm differentiation. Therefore, the mechanisms involving CDKI in differentiation are likely to be much more complex. Indeed, several studies have suggested that KIP/CIP family CDKIs could be located on the chromatin and interfere directly with the transcriptional activity of specific genes. p21 has been shown to bind E2F1 (Delavaine and La Thangue, 1999) while p27 interacts with p130-E2F4 complex to recruit transcriptional co-repressors such as Sin3A and histone deacetylases (Pippa et al., 2012). p27 can also interact with Neurogenin-2 to promote the differentiation of neuronal progenitors in the cortex (Nguyen et al., 2006) while p57 can form a complex with MYOD to promote expression of muscle-specific genes (Reynaud et al., 2000). Therefore, CDKIs could have a function during cell-fate specification as transcriptional regulators besides their role as cell-cycle inhibitors.

Since CDKIs form a regulatory loop with key signaling pathways to promote specification toward the endoderm lineage such regulation could be used in the future for devising a universal method of differentiation that could work with any hIPSCs lines for a diversity of therapeutic applications, disease modeling, or *in vivo* reprogramming (Ladewig et al., 2013; Sterneckert et al., 2014; Tabar and Studer, 2014). The positive feedback regulation of CDKIs could also be exploited for guiding lineage specification of complex tissues during *in vitro* organogenesis (Sasai, 2013; Sterneckert et al., 2014), by controlled expression of distinct CDKIs in a dynamic, stepwise, and lineage-specific manner for deriving organoid systems. Furthermore, the function of CDKIs in aging cells as inducers of senescence and as tumor suppressors (Collado et al., 2007; Kim and Sharpless, 2006), could be particularly relevant for overcoming the current roadblock in regenerative medicine in deriving cell types with adult cell characteristics instead of their immature fetal-like properties (Oh et al., 2014; Tabar and Studer, 2014).

One of the main objectives in biomedicine is using hPSCs to differentiate them into functional cell types such as pancreatic beta-cells for biomedical applications, but the low efficiency of cell differentiation has remained a challenge. The identified positive feedback circuitries among SMAD2/3, CDKIs, and developmental transcription factors such as EOMES could be beneficial for the spontaneous self-organization of cells and tissue formation. To examine the practical utility of our findings, we differentiated hPSCs to endoderm and further to pancreatic lineage by manipulating the activity of SMYD2, EZH2, p18, and CDK4/6 with a small compound that impacted the different stages and efficiency of pancreatic insulin-producing beta-cell differentiation. Hence, our findings have practical significance for more efficient generation of hIPSC-derived cell types for biomedical applications. This could be particularly useful in regenerative medicine and cell replacement therapies by differentiating patient-derived iPSCs to functional cell types such as insulin-producing beta-cells for Type I diabetes patients.

In summary, our results unravel the molecular mechanisms by which cell-fate decisions are controlled by the cell-cycle machinery and describe a dynamical coordination of lineage specification with cell-cycle progression. This knowledge could be utilized for novel strategies in the directed differentiation of stem cells toward clinically relevant cell types as well as for more robust production of functional cells for drug screening and disease modeling. Furthermore, given the function of CDKIs as tumor suppressors, the identification of the regulatory machinery controlling CDKI expression suggests candidate pathways to the deregulation of the cell cycle in tumorigenic processes and cancer stem cells.

## Materials and methods

### Cell culture of hPSCs and FUCCI-hPSCs lines

hPSCs (H9 from WiCell) were grown in defined culture conditions as described previously (Vallier et al., 2009b). H9 cells were passaged weekly using collagenase IV and maintained in chemically defined medium (CDM) supplemented with ACTIVIN A (10 ng/mL) and FGF2 (12 ng/mL). Pluripotent cells were maintained in Chemically Defined Media with BSA (CDM-BSA) supplemented with 10 ng/mL recombinant human ACTIVIN A and 12 ng/mL recombinant human FGF2 (both from Dr. Marko Hyvonen, Department of Biochemistry, University of Cambridge). Cells were passaged every 4–6 days with collagenase IV as clumps of 50–100 cells and dispensed at a density of 100–150 clumps/cm^2^. The culture media was replaced 48 h after the split and then every 24 h. Alternative culture conditions were used to maintain hPSCs by maintaining cells on Vitronectin (StemCell Technologies)-coated plates in Essential 8 (E8) medium (Life technologies). Cells were passaged every 5–7 days using 0.5 μmol/L EDTA and plated onto fresh vitronectin-coated plates in E8 medium. The medium was refreshed every second day. This change corresponds to the modification of protocols in our lab and has no influence on experimental outcomes. The generation of FUCCI-hPSC lines has been described in Pauklin and Vallier (2013) and is based on the FUCCI system described in Sakaue-Sawano et al. (2008).

### *In vitro* differentiation of hPSCs

FUCCI-hPSCs were differentiated into endoderm as described previously (Vallier et al., 2009). Differentiation into endoderm was performed for up to 72 h with a combination of cytokines as described in (Pauklin and Vallier, 2013; Pauklin et al., 2016). For cells sorted by FACS, the cells were collected and immediately placed into the endoderm differentiation media. Endoderm specification was performed in CDM with polyvinyl alcohol (CDM-PVA) prepared SW with 50 ng/mL FGF2, 1 μmol/L Ly-294002 (Promega), 100 ng/mL ACTIVIN A, and 10 ng/mL BMP4 (R&D) for 3 days. Alternatively, and for cells grown in E8 medium, H9 cells were plated as single cells onto gelatin/MEF-coated plates in E8 medium supplemented with 10 μmol/L Y-27632. The medium was refreshed the next day. Chemically defined media with polyvinyl Aalcohol (CDM-PVA) containing 100 ng/mL recombinant ACTIVIN A (CSCR, University of Cambridge), 80 ng/mL FGF2 (R&D Systems), 10 ng/mL BMP4 (CSCR, University of Cambridge), 10 μmol/L LY29004 (Promega), and 3 μmol/L CHIR99021 (Selleck Chemicals) was applied to the cells for 24 h. The media was then replaced with fresh CDM-PVA supplemented with 100 ng/mL recombinant ACTIVIN A (CSCR, University of Cambridge), 80 ng/mL FGF2 (R&D Systems), 10 ng/mL BMP4 (CSCR, University of Cambridge), and 10 μmol/L LY29004 (Promega). The next day, the media was removed and RPMI media supplemented with 1× B27 (Lifetech), 100 ng/mL ACTIVIN A, 80 ng/mL FGF2, and 1× nonessential amino acids (Lifetech) was added to the cells.

### Generating INK4 and KIP/CIP knockdown cells

Previously validated shRNA expression vectors from Sigma-Aldrich (Table S1) were transfected into H9 hPSCs with lipofectamine 2000 (Pauklin and Vallier, 2013) and grown for 3 days. Cells were then cultured in the presence of puromycin until antibiotic-resistant colonies appeared. These were picked and characterized for knockdown efficiency.

### Generating INK4 and KIP/CIP overexpressing cells

For CDKI overexpression, cDNA sequences of *p14*, *p15*, *p16*, *p18*, *p21*, *p27,* and *p57* were transferred into a pTP6 vector containing a CAG promoter. GFP and empty vector were used as controls. All inserts were confirmed by sequencing. Vectors were transfected into H9 hPSCs by lipofection (Pauklin and Vallier, 2013) and grown for 3 days. Thereafter, cells with a stable integration were selected by continuous presence of puromycin. Individual clones were picked, propagated, and used for subsequent analyses.

### qPCR and immunostaining

Methods for Q-PCR and immunostaining have been described previously (Vallier et al., 2009c). Q-PCR data are presented as the mean of three independent experiments and error bars indicate standard deviations. Antibodies and primer sequences are listed in Tables S2 and S3.

### Knockin hPSCs for *OCT4*

H9 hPSCs with *OCT4-eGFP* knockin were obtained from WiCell.

### The small molecule screening library

The screening library contained concentrated small molecule compounds with verified biochemical activity against their targets. Most of the compounds target epigenetic regulators with high specificity (Table S4).

### Screening of the chemical compounds

The cells were grown in 96-well plates in standard growth medium with puromycin (1 µg/mL stock). Three technical replicates and three biological replicates were used for the screening. Cells were plated at a concentration of 10,000 cells in 100 μL of media per well in a 96-well plate. One day after plating the cells, the medium was changed to 90 μL standard growth medium supplemented with puromycin (0.5 µg/mL) and ACTIVIN A (10 ng/mL). On the same day, the compounds were added: first, 100× compound library dilutions were made, and 10 μL of 100× diluted chemical was added to each well to obtain 1000× final dilution of the compounds. Cells were then cultured with chemical compounds for 5 days with media change at Days 0, 2, and 4 supplemented by fresh compounds. Each replicate was analyzed using Celigo Image Cytometer (Nexcelom) and flow cytometry. Cells were lifted and dissociated into single cells with Trypsin. Details on the antibodies that were used for flow cytometry are listed in Table S5. The cells were incubated with 0.5 μg/mL final concentration of conjugated antibodies in 1% BSA-PBS for 40 min on ice and washing was repeated as before. The cells were then suspended in 300 μL 1% BSA-PBS with DAPI (1:2,000) for live/dead separation and kept on ice to be used for the flow cytometry analysis.

### Gene knockdown and overexpression

The stable knockdown and overexpression of SNON was performed by using previously published constructs (Tsuneyoshi et al., 2012) kindly provided by Prof. Ray Dunn at the Institute of Medical Biology, A*STAR (Agency for Science, Technology, and Research), Singapore. shRNA plasmid DNA was transfected into cells with Lipofectamine 3000 (Thermo Fischer Scientific) according to manufacturer guidelines. Puromycin was added to the growth media at 0.1 μg/mL concentration and individual colonies were picked, expanded, and screened for gene knockdown compared to Scramble control transfected cells.

### Nucleic acid extraction from cell lines

RNA was extracted using Direct-zol (TM) RNA extraction kit according to manufacturer protocol (Cambridge Bioscience, R2052). The quality of the RNA samples was verified using an RNA screen tape on a Tape-Station (Agilent). The RIN values for all samples were >7.5.

### RNA isolation and cDNA synthesis

Total RNA was isolated by RNeasy RNA Extraction Kit (Qiagen) according to the manufacturer’s guidelines. RNA was then eluted in 30 μL of water and the concentration was measured using Nanodrop. The master mix was prepared as follows: 8 μL 5× First-Strand Buffer (Invitrogen), 0.5 μL Random primers (0.5 μg/mL) (Promega Cat. C1181), 1 μL dNTP mix (10 mmol/L each) (Promega Cat.U1515), 2 μL 0.1 mol/L DTT, 0.5 μL RNase Out, 0.25 μL Superscript III Reverse Transcriptase (Life Technologies), 500 ng of total RNA into a separate tube with 11.75 μL RNase-free water. RNA was heated to 65°C for 5 min and allowed to chill on ice for 2 min. A total of 8.25 μL of the master mix was added to RNA. The reaction was incubated at 25°C for 10 min and then at 42°C for 50 min. The reaction was then inactivated by heating at 70°C for 15 min.

### RT-qPCR

Synthesized cDNA (2 ng) was added to 5 μL Power SYBR Mix (Life Technologies, 4368708 (Master Mix)) and 1.5 μL, 2 μmol/L of forward and reverse primers. RT-qPCR was performed on ViiA 7 machine with the following intervals: denaturation (95°C) for 15 s and a total of 40 cycles, annealing/extension (60°C) for 60 s, and final extension (60°C) for 10 min.

### Flow cytometry for cell-cycle analysis

Cells were collected and analyzed using Fortessa (BD Bioscience). Passaging was performed a Day 5, after which cells were plated again in spheroid conditions, with the same initial density of 5,000 cells/1 mL medium. Compounds were added on Day 7, and the treatment lasted for 72 h. Experiment was performed in three replicates. The data was analyzed in FlowJo.

### Western blot analysis

Protein was isolated by lysing cells with RIPA Buffer (Sigma-Aldrich) supplemented by cOmplete EDTA-free protease inhibitor (Roche) and PhosSTOP^™^ (Sigma-Aldrich) and extracting the supernatant after high-speed centrifugation at 4°C. Protein quantification was performed using the Pierce BCA Protein Assay kit following the manufacturer’s protocol. Isolated proteins were prepared for SDS-PAGE separation by dilution with 4× NuPAGE Sample buffer (Invitrogen), addition of NuPAGE™ Sample Reducing Agent [(10×), Invitrogen], 95°C for 5 min, and cooling. Isolated proteins were then analyzed by Western blot. Protein separation via SDS-PAGE was performed on a NuPAGE 4%–12% or 12% Bis-Tris gel (Life Technologies) with NuPAGE™ MOPS SDS Running Buffer (Life Technologies). Proteins were transferred to a PVDF membrane, blocked with 5% milk in PBS and 0.05% Tween-20, probed with protein-specific antibodies, incubated with horseradish peroxidase-conjugated secondary antibodies, and visualized via enhanced chemiluminescence using the SuperSignal West Pico Chemiluminescent Substrate (Thermo Scientific). All antibodies (Table S6) were diluted in 5% milk in PBS and 0.05% Tween-20. Quantification was performed using ImageJ gel analysis tool.

### Immunostaining

The immunostaining method has been described previously (Bertero et al., 2015; Pauklin and Vallier, 2013; Pauklin et al., 2016). Cells were fixed for 20 min at 4°C in PBS 4% PFA (electron microscopy grade), rinsed three times with PBS, then blocked and permeabilized at the same time for 30 min at room temperature using PBS with 10% Donkey Serum (Biorad) and 0.1% Triton X-100 (Sigma). Incubation with primary antibodies diluted in PBS 1% Donkey Serum and 0.1% Triton X-100 was performed overnight at 4°C. Samples were washed three times with PBS, and then incubated with AlexaFluor secondary antibodies for 1 h at room temperature protected from light. Cells were finally washed three times with PBS, and Hoechst (Sigma) was added to the first wash to stain nuclei. Images were acquired using an LSM 700 confocal microscope (Leica).

### Chromatin immunoprecipitation (ChIP)

All steps were performed on ice or at 4°C and ice-cold buffers and PBS were supplemented with 1 mg/mL Leupeptin, 0.2 mmol/L PMSF, and 10 mmol/L NaButyrate were used unless otherwise stated. Approximately 5 × 10^6^ cells were used per sample and cross-linked with 1% formaldehyde for 15 min. Cross-linking was stopped by incubating samples with glycine at a final concentration of 0.125 mol/L for 5 min at room temperature, and the cells were washed with PBS followed by pelleting at 250 ×*g* for 5 min. The pellet was re-suspended in 2 mL ChIP Cell Lysis Buffer (CLB: 10 mmol/L Tris–pH 8, 10 mmol/L NaCl, 0.2% NP-40) and incubated for 10 min to lyse the plasma membranes. Nuclei were pelleted at 600 ×*g* for 5 min, lysed in 1.25 mL of ChIP Nuclear Lysis Buffer (NLB: 50 mmol/L Tris–pH8, 10 mmol/L EDTA, 1% SDS) for 10 min, and then 0.75 mL of ChIP Dilution Buffer (DB: 20 mmol/L Tris–pH 8, 2 mmol/L EDTA, 150 mmol/L NaCl, 0.01% SDS, 1% Triton X-100) was added to the samples. Chromatin was sonicated in 15 mL Diagenode Bioruptor Pico water bath sonicator with an automated water cooling system, by performing 30 cycles of 30 s ON, 45 s OFF. This protocol resulted in the homogeneous generation of fragments of 100–400 bp. Samples were clarified by centrifugation at 16,000 ×*g* for 10 min, and diluted with 3.5 mL of DB. After preclearing with 10 µg of non-immune IgG for 1 h and 50 μL of Protein G-Agarose for 2 h, ChIP was performed overnight in rotation using specific antibodies (Table S2) or non-immune IgG as a control. After incubation for 1 h with 30 μL of Protein G-Agarose, beads were washed twice with ChIP Washing Buffer 1 (WB1: 20 mmol/L Tris–pH 8, 2 mmol/L EDTA, 50 mmol/L NaCl, 0.1% SDS, 1% Triton X-100), once with ChIP Washing Buffer 2 (WB2: 10 mmol/L Tris–pH 8, 1 mmol/L EDTA, 0.25 mol/L LiCl, 1% NP-40, 1% Deoxycholic acid), and twice with Tris–EDTA (TE: 10 mmol/L Tris–pH 8, 1 mmol/L EDTA). Precipitated DNA was eluted with 150 μL of ChIP Elution Buffer (EB: 100 mmol/L NaHCO_3_) twice for 15 min at room temperature in rotation, and processed as follows in parallel with 300 μL of sonicated chromatin non-used for ChIP (Input). Cross-linking was reverted by adding NaCl to a final concentration of 300 mmol/L for protein-DNA de-crosslinking and incubated at 65°C for 5 h and 1 µg RNase A (Sigma) to digest contaminating RNA. Finally, 60 µg of Proteinase K (Sigma) was added overnight at 45°C. DNA was extracted by sequential phenol–chloroform and chloroform extractions and precipitated overnight at −80°C in 100 mmol/L NaAcetate, 66% ethanol, and 50 µg of glycogen (Ambion) as a carrier. After centrifugation at 16,000 ×*g* for 1 h at 4°C, DNA pellets were washed once with ice-cold 70% ethanol, and finally air dried. ChIP samples were resuspended in 30 μL and 1:10 of the samples were used in qPCR for verifying the ChIP samples. Primers used for CHIP experiments are listed in Table S7.

### Cell fractionations

Cells were harvested with trypsin and washed twice with cold PBS. For cytoplasmic lysis, cells were suspended in five times packed cell volume (1 μL PCV = 10^6^ cells) equivalent to Isotonic Lysis Buffer (10 mmol/L Tris–HCl, pH 7.5, 3 mmol/L CaCl, 2 mmol/L MgCl_2_, 0.32 mol/L sucrose, complete protease inhibitors, and phosphatase inhibitors), and incubated for 12 min on ice. Triton X-100 was added to a final concentration of 0.3% and incubated for 3 min. The suspension was centrifuged for 5 min at 1,500 rpm at 4°C and the supernatant (cytoplasmic fraction) was transferred to a fresh chilled tube. For nuclear lysis, nuclear pellets were resuspended in 2× PCV Nuclear Lysis Buffer + Triton X-100 (50 mmol/L Tris–HCl, pH 7.5, 100 mmol/L NaCl, 50 mmol/L KCl, 2 mmol/L MgCl_2,_ 1 mmol/L EDTA, 10% glycerol, 0.3% Triton X-100, Complete protease inhibitors, and phosphatase inhibitors) and dounce homogenized. The samples were incubated with gentle agitation for 30 min at 4°C and then centrifuged with a Ti 70.1 rotor at 22,000 rpm for 30 min at 4°C or with a Ti 45 rotor for 30 min at 20,000 rpm at 4°C. The chromatin pellets were dounce homogenized in 2× PCV nuclear lysis buffer + Triton X-100 and benzonase until the pellets gave much less resistance. The samples were incubated at RT for 30 min and centrifuged with either a Ti 70.1 rotor for 30 min at 22,000 rpm at 4°C or with a Ti 45 rotor for 30 min at 20,000 rpm at 4°C.

### Protein co-immunoprecipitation

Samples were incubated with 5 μg of cross-linked antibodies for 12 h at 4°C. Beads were washed five times with 10 bead volumes of nuclear lysis buffer and eluted in SDS Western blot buffer (30 mmol/L Tris pH 6.8, 10% glycerol, 2% SDS, 0.36 mol/L beta-mercaptoethanol (Sigma), and 0.02% bromophenol blue) by heating at 90°C for 5 min. Samples were analyzed by standard Western blot techniques.

### Flow cytometry

Flow cytometry was carried out with a BD MoFlo flow cytometer and analyzed by FloJo software. Cell-cycle distribution was analyzed by Click-It EdU incorporation Kit (Invitrogen) according to the manufacturer’s guidelines. Marker expression was analyzed at various timepoints during differentiation by first dissociating cells into single cells with Cell Dissociation Buffer (Gibco) and fixing in 4% PFA for 20 min at 4°C. This was followed by permeabilization and blocking with 10% serum + 0.1% Triton X-100 in PBS for 30 min at RT and incubation with primary antibody in 1% serum + 0.1% Triton X-100 for 2 h at 4°C. After washing the samples three times with PBS, they were incubated with a secondary antibody for 2 h at 4°C, washed three times with PBS, and analyzed by flow cytometry.

### Cell sorting by FACS

FACS on FUCCI-hPSCs was performed as described before (Sakaue-Sawano et al., 2008; Pauklin and Vallier, 2013). hPSCs were washed with PBS and detached from the plate by incubating them for 10 min at 37°C in a cell dissociation buffer (Gibco). Cells were then washed with a cold filter and sterilized with 1% BSA in PBS, before incubating cells in PBS 1% BSA with Tra-1-60 primary antibody (1:100) and Alexa Fluor 647 donkey α-mouse secondary antibody (1:1000) on ice for 20 min in the dark with occasional gentle mixing. The cells were then washed once with at least 50× pellet volume PBS 1% BSA, resuspended gently in 3 mL sterile maintenance media, and subjected to cell sorting by gating Tra-1-60^+^ cells according to the mAG/mKO2 FUCCI signals for hPSCs or mAG/mRFP/mKate2 FUCCI signals for PDAC cells. The cell sorting was performed with a BeckmanCoulter MoFlo MLS high-speed cell sorter by using parameters described previously^18^, and the cells were sorted directly into collection tubes with 2 mL maintenance media.

### Teratoma assays

One million hESC were injected in the lumen of the testicle of 6–8-week-old SCID mice and three animals were injected in each group. After 12 weeks, mice were sacrificed, and the testicles and tumors were dissected and fixed for 48 h in Bouins solution (Sigma-Aldrich). The fixed tissues were then paraffin-embedded and processed according to standard procedures. Sections (5 µm) were stained with hematoxylin/eosin and subsequently examined under a bright-field microscope for the presence of tissues deriving from the three germ layers. Animal procedures were performed in accordance with the local committee on Animal Experimentation at Centro de Investigación Príncipe Felipe.

### Luciferase assay

Cells were transfected with a SMAD2/3 reporter construct (SBE4-luciferase), SOX17 or GSC promoter constructs (Brown et al., 2011), and Renilla luciferase at a ratio of 10:1, using Lipofectamine 2000 (Invitrogen) (Pauklin and Vallier, 2013). Luciferase activity was measured with the dual luciferase assay kit following (Promega) manufacturer instructions. Firefly luciferase activity was normalized to Renilla luciferase activity for cell numbers and transfection efficiency. Samples were analyzed on a Glomax Luminometer and software.

### EdU incorporation assay

Cell-cycle distribution was analyzed by Click-It EdU incorporation Kit (Invitrogen) according to the manufacturer’s guidelines. Flow cytometry was carried out with a BD MoFlo flow cytometer and analyzed by FloJo software. Cells were cultured in media collected from cells with different treatment conditions for 72 h, replacing the media every 24 h.

### Statistical analysis

GraphPad Prism 6 was used for statistical analysis by performing *t*-test and two-way ANOVA tests followed by Bonferroni’s corrected multiple comparisons between pairs of conditions. Unless otherwise indicated in the figure legends, we analyzed three biological replicates for each data point in all graphs, and the level of significance was as follows: *P* < 0.1 (*), *P* < 0.05 (**), *P* < 0.01 (***), and *P* < 0.001 (****).

## Supplementary data

The online version contains supplementary material available at https://doi.org/10.1093/procel/pwae031.

pwae031_suppl_Supplementary_Tables_S1-S6_Figures_S1-S11

## Data Availability

Further information and requests for resources and reagents should be directed to and will be fulfilled by the lead contact, Siim Pauklin (siim.pauklin@ndorms.ox.ac.uk). Newly generated materials associated with the paper should be requested by contacting the lead contact.

## Code availability

All data analyses were followed by standard programs. This paper does not report original code.
