# Supplementary material for: SMAD2/3-SMYD2 and developmental transcription factors cooperate with cell-cycle inhibitors to guide tissue formation
Source: Protein Cell. 2024 May 17;16(4):259–82. doi: 10.1093/procel/pwae031 (PMC12053477; doi:10.1093/procel/pwae031)

Supplementary Information

SMAD2/3-SMYD2 and developmental transcription factors cooperate with cell cycle inhibitors to guide tissue formation

Supplementary Table 1. shRNA constructs used for CDKI knockdown.

Gene name	ShRNA construct 1	ShRNA construct 2	ShRNA construct 3
P14/p16	TRCN0000255849	TRCN0000255853	TRCN0000281415
P15	TRCN0000262323	TRCN0000262324	TRCN0000262325
P18	TRCN0000037595	TRCN0000037597	TRCN0000226407
P21	TRCN0000287021	TRCN0000287091	TRCN0000294421
P27	TRCN0000009856	TRCN0000009857	TRCN0000039930
P57	TRCN0000237879	TRCN0000237878	TRCN0000368957

Supplementary Table 2. Antibodies.

**Antibody specificity**	**Techniques**	**Catalogue name**	**Company**
NANOG	IF, WB, IP	AF1997	R&D Systems
Oct4	IF, WB	sc-5279	Santa Cruz Biotechnology
Sox2	IF, WB	AF2018	R&D Systems
Eomes	IF, WB, CHIP, IP	ab23345	Abcam
Brachyury	IF, WB	AF2085	R&D Systems
Sox17	IF, WB	AF1924	R&D Systems
Pax6	IF, WB	PRB-278P-100	Cambridge BioScience
Sox1	IF, WB	AF3369	R&D Systems
Actin	WB	MAB1501	Chemicon
Histone H3	CHIP	ab1791	Abcam
Histone H3 (tri methyl K4)	CHIP	ab8580	Abcam
Histone H3 (tri methyl K27)	CHIP	ab6002	Abcam
Histone H3 (tri methyl K27)	CHIP	07-449	EMD Millipore
Histone H3 (mono	CHIP	ab8895	Abcam

methyl K4)			
Histone H3 (acetyl K27)	CHIP	ab4729	Abcam
Histone H3 (tri methyl K9)	CHIP	ab8898	Abcam
Histone H3 (tri methyl K36)	CHIP	ab9050	Abcam
SMAD2/3	IP, WB, CHIP	AF3797	R&D Systems
CXCR4	IF, FACS	MAB173	R&D Systems
Tra-1-60	IF, FACS	sc-21705	Santa Cruz
p14 ARF (C-18)	IF, WB	sc-8613	Santa Cruz Biotechnology
p15 (C-20)	IF, WB	sc-612	Santa Cruz Biotechnology
p16 (C-20)	IF, WB	sc-468	Santa Cruz Biotechnology
p18 (N-20)	IF, WB	sc-865	Santa Cruz Biotechnology
p21 (C-19)	IF, WB	sc-397	Santa Cruz Biotechnology
p27 (C-19)	IF, WB	sc-528	Santa Cruz Biotechnology
p57 (C-20)	IF, WB	sc-1040	Santa Cruz Biotechnology
BAZ2A/TIP5	WB, CHIP	Ab195278	Abcam
SUV39H1	WB, CHIP	C15410368	Diagenode
SAP18	IP, WB, CHIP	13841-1-AP	Proteintech
SAP18	IP, WB, CHIP	Ab175920	Abcam
SNW1	IP, WB, CHIP	25926-1-AP	Proteintech
EZH2 (D2C9)	WB, CHIP	5246S	Cell Signalling Technology
5-mC	CHIP	C15200006	Diagenode
Alexa Fluor 647 goat α-mouse IgM	IF, FACS	A21238	Invitrogen
Alexa Fluor 647 donkey α-mouse	IF, FACS	A31571	Invitrogen
Alexa Fluor 647 donkey α-goat	IF, FACS	A21447	Invitrogen

Supplementary Table 3. Primers used in Q-PCR.

Primer name	Primer Sequence
PBGD	F GGAGCCATGTCTGGTAACGG<br>R CCACGCGAATCACTCTCATCT
NANOG	F CATGAGTGTGGATCCAGCTTG<br>R CCTGAATAAGCAGATCCATGG
OCT4	F AGTGAGAGGCAACCTGGAGA<br>R ACACTCGGACCACATCCTTC
Sox2	F TGGACAGTTACGCGCACAT<br>R CGAGTAGGACATGCTGTAGGT
Eomes	F ATCATTACGAAACAGGGCAGGC<br>R CGGGGTTGGTATTTGTGTGTAAGG
Gsc	F GAGGAGAAAGTGGAGGTCTGGTT<br>R CTCTGATGAGGACCGCTTCTG
Sox17	F CGCACGGAATTTGAACAGTA<br>R GGATCAGGGACCTGTCACAC
Brachyury T	F TGCTTCCCTGAGACCCAGTT<br>R GATCACTTCTTTCCTTTGCATCAAG
Mesp1	F GAAGTGGTTCCTTGGCAGAC<br>R TCCTGCTTGCCTCAAAGTGT
Sox1	Sigma Quantitect primers
Pax6	F CTTTGCTTGGGAAATCCGAG<br>R AGCCAGGTTGCGAAGAACTC
p14	GAACATGGTGCGCAGGTTCTTGGT<br>CTCGGGCGCTGCCCATCATC
p15	ATCGCCGATGTAGATTTGTACAGG<br>CCAAGTCCACGGGCAGACG
p16 INK4	GAGGAGGGGCTGGCTGGTCA<br>TGCACGGGTCGGGTGAGAGT
P18	CACCGAACTGCTACTCCCGT<br>AACTCGTTCCCCAAGGCTC
p21	CATGTGGACCTGTCACTGTCTTGTA<br>AGACTAAGGCAGAAGATGTAGAGCG
p27	AAGCCTGGCCTCAGAAGACG<br>ATGTCCATTCCATGAAGTCAGCG

p57	GCTAGCCAGCAGGCATCGAG<br>GTACTGGGAAGGTCCCACGG

Supplementary Table 4. Small molecule compounds used in the screening experiment. List with compound names, working concentrations and target molecules.

Vial	Source Name	[Working] uM	Class/Target
1	(+)-JQ1	1	Bromodomains - BRD2, BRD3, BRD4, BRDT (BET)
2	(-)-JQ1 (inactive)	1	Bromodomains - Negative control
3	PFI-1	5	Bromodomains - BRD2, BRD3, BRD4, BRDT (BET)
4	I-BET	1	Bromodomains - BRD2/3/4
5	Bromosporine	1	Bromodomains - pan-Bromodomain
6	CBP/BRD4 (0383)	5	Bromodomains - CBP, BRD4(1)
9	SGC-CBP30	1	Bromodomains - CREBBP, EP300
10	I-CBP112	1	Bromodomains - CREBBP, EP300
15	RVX-208	5	Bromodomains - BRD2, BRD3, BRD4, BRDT (BET, BD2)
16	SMARCA	2.5	Bromodomains - SMARCA, PB1
17	PB1/SMARCA	1	Bromodomains - SMARCA, PB1
18	PFI-3	1	Bromodomains - SMARCA2/4, PB1(5)
19	GSK2801	1	Bromodomains - BAZ2A, BAZ2B
20	PFI-4	1	Bromodomains - BRPF1B
21	TRIM24/BRPF	10	Bromodomains - TRIM24/BRPF
22	OF-1	5	Bromodomains - pan-BRPF
23	Belinostat	5	HDAC - hydroxamic acids
24	CXD101	1	HDAC -

25	Valproic acid	1000	HDAC - aliphatic acid compounds
26	Entinostat	0.5	HDAC - ortho-amino anilides
27	SAHA	2.5	HDAC - hydroxamic acids
28	Trichostatin A	0.5	HDAC - hydroxamic acids - Class I & II
29	SRT1720	1	HDAC - SIRT1 (indirect?) activator
30	EX 527	1	HDAC - SIRT1
32	CI-994	1	HDAC - 1,2,3,(8)
33	CPI-360	10	Histone methyltransferase - EZH2 and EZH1
34	CPI-413	10	Histone methyltransferase - EZH2 and EZH1
35	UNC0638	1	Histone methyltransferase - G9a, GLP
37	UNC0642	1	Histone methyltransferase - G9a, GLP
38	A-366	2	Histone methyltransferase - G9a, GLP
39	Chaetocin	0.05	Histone methyltransferase - SUV39H1
43	PFI-2	2	Histone methyltransferase - SETD7
45	SGC0946	7.5	Histone methyltransferase - DOT1L
46	GSK343	3	Histone methyltransferase - EZH2
47	UNC1999	1	Histone methyltransferase - EZH2
49	LLY-507	1	Histone methyltransferase - SMYD2
50	Tranylcypromine	20	Lysine demethylases - LSD1
51	GSK-LSD1 (irreversible)	0.5	Lysine demethylases - LSD1
52	GSK690	5	Lysine demethylases - LSD1
53	GSK J4	10	Lysine demethylases - JMJD3, UTX, JARID1B
54	GSK J5 (inactive)	10	Lysine demethylases - Negative control
55	IOX1 (5-carboxy-	40	Lysine demethylases - pan-2-OG

	8HQ)		
57	Methylstat (Ester)	2.5	Histone demethylase
58	(E)-JIB-04	0.05	Histone demethylase - Pan JmjC
60	ML324	5	Histone demethylase - JMJD2E
61	IOX2	10	Prolyl-Hydroxylases - PHD2 (EGLN1)
62	OICR-9429	1	Methyl Lysine Binder? - WDR5
63	UNC1215	5	Methyl Lysine Binder - L3MBTL3
64	5-Azacitidine	10	DNA methyltransferase (DNMT) -
65	5-Azadeoxycitidine	5	DNA methyltransferase (DNMT) - DNMT1/3
66	Olaparib	1	Poly ADP ribose polymerase (PARP)
67	Rucaparib	10	Poly ADP ribose polymerase (PARP)
68	K00135	1	Kinase inhibitor - ATP competitive - PIM
69	5-Iodotubercidin	1	Kinase inhibitor - ATP mimetic - Haspin
70	C646	1	Histone acetyltransferase (HAT) p300/CBP
74	DUAL1946	1	
75	GSK484	1	Peptidyl arginine deiminase (PAD4)
76	KDOBA67	10	Histone demethylase
77	BAZ2-ICR	1	Bromodomains - BAZ2A, BAZ2B
78	NI-57	1	Bromodomains - pan-BRPF
79	LP99	1	Bromodomains - BRD9, BRD7
80	SGC707	1	Arginine methyltransferase - PRMT3
81	RGFP966	10	HDAC - HDAC3
82	PCI-34051	5	HDAC - HDAC8
83	Rocilinostat	10	HDAC - HDAC6
84	Tubastatin A HCl	10	HDAC - HDAC6

85	KDOAM-25a	1	Lysine demethylases - JARID
86	KDM5-C70	10	Histone demethylase - JARID1
88	MAZ1805	1	t-RNA sythetase
89	MAZ1392	1	t-RNA sythetase
90	BI-9564	1	Bromodomains - BRD9, BRD7
91	NVS-CECR2-1	1	Bromodomains - CECR2
92	GSK106	1	Peptidyl arginine deiminase (PAD4)
93	J556-42R	1	Arginine methyltransferase - PRMT5
94	J556-63R	1	Arginine methyltransferase - PRMT5
95	J556-70R	1	Arginine methyltransferase - PRMT5
96	A-196	1	Histone methyltransferase - SUV420H1/H2
97	BAY-598	1	Histone methyltransferase - SMYD2
98	J556-143	1	Arginine methyltransferase - PRMT5
99	MS049	1	Arginine methyltransferase
100	MS023	1	Arginine methyltransferase - Type I PRMTs
101	MS003	1	Arginine methyltransferase - negative control
104	SGI-1776	10	Kinase inhibitor - Haspin
105	CHR-6494	1	Kinase inhibitor - Haspin
106	CPI-169	10	Histone methyltransferase - EZH2, EZH1
107	UNC2400	1	Histone methyltransferase - EZH2
108	GSK864	5	Dehydrogenase
109	GSK8814	10	Bromodomains - ATAD2
110	GSK8815	10	Bromodomains - ATAD2
111	GSK959	1	Bromodomains - BRPF1
112	NVS-CECR2-C	1	Bromodomains - CECR2

113	BAY-299	1	Bromodomains - BRD1, TAF1
114	PCI-24781	10	HDAC -
115	Romidepsin	1	HDAC -
116	Mocetinostat	10	HDAC -
117	Santacruzamate	50	HDAC 2?
118	KDOAM32	1	Lysine demethylases - JARID
120	MS409N	1	Arginine methyltransferase - PRMT4, PRMT6 inactive control
121	TP-064	1	Arginine methyltransferase - PRMT4
122	TP-064N	1	Arginine methyltransferase - PRMT4
123	A-395	1	Methyl Lysine Binder - EED
124	A-395N	1	Methyl Lysine Binder - EED
125	I-BRD9	10	Bromodomains - BRD9
126	TP-472	1	Bromodomains - BRD9
127	TP-472N	1	Bromodomains - BRD9
128	KDOPZ-32a	1	Lysine demethylases - KDM5
129	KDOOA012000	1	Lysine demethylases KDM2
130	AMI-1	50	Arginine methyltransferase - PRMT
131	TMP269	10	HDAC -4, 5, 7 &9
132	AGK2	10	HDAC - SIRT2
133	GSK6853	1	Bromodomains - BRPF1/2/3
134	GSK9311	1	Bromodomains - BRPF1/2/3
135	LLY-283	1	Arginine methyltransferase - PRMT5
136	TD001851a	1	Methyl Lysine Binder/tudor domain -Spin1
137	TDOSI000058a	1	Methyl Lysine Binder/tudor domain -Spin1

138	TD001863a	1	Methyl Lysine Binder/tudor domain -Spin1
139	TDOSI000062a	1	Methyl Lysine Binder/tudor domain -Spin1
140	TD001857a	1	Methyl Lysine Binder/tudor domain -Spin1
141	TD001856a	1	Methyl Lysine Binder/tudor domain -Spin1
142	TD001858a	1	Methyl Lysine Binder/tudor domain -Spin1
143	TMP195	1	HDAC -4,5,7,9
144	GSK2879552	10	Lysine demethylases - LSD1
145	TDO20821a	1	Methyl Lysine Binder/tudor domain -Spin1
146	TDO20824a	1	Methyl Lysine Binder/tudor domain -Spin1
147	TDO20823a	1	Methyl Lysine Binder/tudor domain -Spin1
148	A-485	1	Histone acetyltransferase (HAT) p300/CBP
149	A-486	1	Histone acetyltransferase (HAT) p300/CBP
150	GSK4027	1	Bromodomains - PCAF, GCN5
151	GSK4028	1	Bromodomains - PCAF, GCN5
152	L-Moses	1	Bromodomains - PCAF, GCN5
153	D-Moses	1	Bromodomains - PCAF, GCN5
154	PFI-5	1	Histone methyltransferase - SMYD2
155	YX39-31b	1	Methyl Lysine Binder/tudor domain -Spin1
156	TDO208229	1	Methyl Lysine Binder/tudor domain -Spin1
157	TDO01856a	1	Methyl Lysine Binder/tudor domain -Spin1
158	TDO20826a	1	Methyl Lysine Binder/tudor domain -Spin1
159	Bortezomib	0.1	Protesome
160	Carfilzomib	0.1	Protesome
161	RTS-V5	1	Protesome and HDAC
162	dBRD9	1	Bromodomains - BRD9

163	BI-7273	0.1	Bromodomains - BRD9/7 (IC50 19/117nM)
164	CPI-621	0.1	Lysine demethylases - KDM5

Supplementary Table 5. Antibodies used for the detection of GFP-OCT4, CD133 and SSEA4 by flow cytometry in the compound screening experiment.

Antibody	Target/function	Ratio	Number of cells
CD133-BV786, Mouse Anti-Human, clone W6B3C1, BD 747640	CD133	0.6 uL in 100 uL	300,000
Mouse IgG1-BV786l, BD 563330	isotype control for CD133	0.6 uL in 100 uL	300,000
Alexa647 Mouse anti-SSEA-4 clone MC813-70	SSEA4	2.5 uL to 100 uL	300,000
Alexa647 Mouse IgG3	isotype control for SSEA4	0.31 uL to 100 uL	300,000

Supplementary Table 6. Primers used in Chromatin Immunoprecipitation. ChIP-qPCR primers were designed to span the regions of SMAD2/3 peaks in hPSCs obtained by SMAD2/3 ChIP-sequencing. These genomic regions overlap with OCT4 binding peaks in hPSCs.

Gene	Primer name	Protein CHIP	Sequence
EOMES	C_eomes1aF	SMAD2/3, OCT4, NANOG, SNON, H3K4me3, H3K27me3 CHIP	GTTCCGGCCGATTACTTGTC
	C_eomes1aR	SMAD2/3, OCT4, NANOG, SNON, H3K4me3, H3K27me3 CHIP	GGGAGGCTTCCGACTACTCA
Mixl1	C_MixL11F	SMAD2/3, OCT4, NANOG, SNON, H3K4me3,	TTTGATGAGGACAGACGGGA

		H3K27me3 CHIP	
	C_MixL11R	SMAD2/3, OCT4, NANOG, SNON, H3K4me3, H3K27me3 CHIP	CGAAGGACTATTTGCCTGGG
GSC	C_GSC1F	SMAD2/3, OCT4, NANOG, SNON, H3K4me3, H3K27me3 CHIP	GTGCAGGGCACAGTTCAGAG
	C_GSC1R	SMAD2/3, OCT4, NANOG, SNON, H3K4me3, H3K27me3 CHIP	TATGGGACGCTTTGAATCCC
Neg Control	SMAD7-veF	SMAD2/3, OCT4, NANOG, SNON, H3K4me3, H3K27me3 CHIP	ACCCTGATAGGAAGAGGGGAAG
	SMAD7-veR	SMAD2/3, OCT4, NANOG, SNON, H3K4me3, H3K27me3 CHIP	TCACACACACTCCTGACAAGTGA
P15	P15 Prom F	SMAD2/3, OCT4, NANOG, SNON, H3K4me3, H3K27me3 CHIP	GCCATCCTCTTTTTCTTCAGCG
	P15 Prom R	SMAD2/3, OCT4, NANOG, SNON, H3K4me3, H3K27me3 CHIP	CCTCCTTGTCTTGAATTTTCGCA
P14/16	P16 Prom F	SMAD2/3, OCT4, NANOG, SNON, H3K4me3, H3K27me3 CHIP	ATAAGGCTCTTCCTTCCTCGG
	P16 Prom R	SMAD2/3, OCT4, NANOG, SNON, H3K4me3, H3K27me3 CHIP	GGCAAGGTTAGGTCGCTGTT
P18	P18 Prom F	SMAD2/3, OCT4, NANOG, SNON, H3K4me3, H3K27me3 CHIP	CATTTTGCCTGTCATCGAGGT
	P18 Prom R	SMAD2/3, OCT4, NANOG, SNON, H3K4me3, H3K27me3 CHIP	CGAGACTTGAAGCTTCCGCA

P21	P21 Prom F	SMAD2/3, OCT4, NANOG, SNON, H3K4me3, H3K27me3 CHIP	CCCAGCATCCCTTCCACTCTTC
	P21 Prom R	SMAD2/3, OCT4, NANOG, SNON, H3K4me3, H3K27me3 CHIP	CATCCTCCTGGGAGCTGGTTTA
P21	P21 Intronic F	SMAD2/3, OCT4, NANOG, SNON, H3K4me3, H3K27me3 CHIP	AGCTTTCACCCCCAGAAACT
	P21 Intronic R	SMAD2/3, OCT4, NANOG, SNON, H3K4me3, H3K27me3 CHIP	CCCTTCAGGAGAGGGAAAAC
P27	P27 Prom F	SMAD2/3, OCT4, NANOG, SNON, H3K4me3, H3K27me3 CHIP	TGGTCTCTTGGTGCATCTGT
	P27 Prom R	SMAD2/3, OCT4, NANOG, SNON, H3K4me3, H3K27me3 CHIP	ATACAAGCAGTGTGAAGGGCA
P57	P57 Prom F	SMAD2/3, OCT4, NANOG, SNON, H3K4me3, H3K27me3 CHIP	CTCCCACTTTGACCTGGTCCC
	P57 Prom R	SMAD2/3, OCT4, NANOG, SNON, H3K4me3, H3K27me3 CHIP	TTATCCCCTGGTCTGGTGGG
Oct4	Oct4 K36me3 F	H3K36me3 CHIP	GCACACCCTGGCACCCCTTG
	Oct4 K36me3 R	H3K36me3 CHIP	TCAGCAGGGCTGGATGCCTT
NANOG	NANOG K36me3 F	H3K36me3 CHIP	CCTGGGAAGCGTTGACCCAACT
	NANOG K36me3 R	H3K36me3 CHIP	CCCCACCCCTTGCAGCTACC
Sox2	Sox2 K36me3 F	H3K36me3 CHIP	CCCTGGCATGGCTCTTGGCT
	Sox2 K36me3 R	H3K36me3 CHIP	TCGGCGCCGGGGAGATACAT
EOMES	EOMES K36me3 F	H3K36me3 CHIP	CGGCTCTTGCAGGGTGCCAA
	EOMES K36me3 R	H3K36me3 CHIP	ACAAGCATACCGCCGAGCAGG
Sox17	Sox17 K36me3 F	H3K36me3 CHIP	TGCAGGCAAGTCGTGGAAGGC
	Sox17 K36me3 R	H3K36me3 CHIP	ACCCGCTTCAGCCGCTTCAC

Mixl1	Mixl1 K36me3 F	H3K36me3 CHIP	TGTCTGTCTGTAGCCCCGTGGT
	Mixl1 K36me3 R	H3K36me3 CHIP	AGGAGGTATTTTGCTCATGCCAGC
GSC	GSC K36me3 F	H3K36me3 CHIP	TGCTGGAGGGCGCTTTAGGC
	GSC K36me3 R	H3K36me3 CHIP	ATCAAAGGCGCGCTTCCCCC
Sox1	Sox1 K36me3 F	H3K36me3 CHIP	CCAAGTGACGCGGAGCTCGT
	Sox1 K36me3 R	H3K36me3 CHIP	GCCGTCTGAAGGAGGGGGTG
Pax6	Pax6 K36me3 F	H3K36me3 CHIP	AGGCCACGCTGTCCTGGAGT
	Pax6 K36me3 R	H3K36me3 CHIP	CTGCTGCCGCGAACTTGAGC
T	T K36me3 F	H3K36me3 CHIP	GTCGTGGCAGCCAGTGGTGA
	T K36me3 R	H3K36me3 CHIP	TGAGCAAGGGATGCTGGGGC
P14/p16	P14/P16 K36me3 F	H3K36me3 CHIP	CCGCTTCTGCCTTTTCACTG
	P14/P16 K36me3 R	H3K36me3 CHIP	CCCTGAGCTTCCCTAGTTCAC
P15	P15 K36me3 F	H3K36me3 CHIP	CCCACAACTTAGGCCCTAGC
	P15 K36me3 R	H3K36me3 CHIP	GGCTTCCAGAGAGTGTCGTT
P18	P18 K36me3 F	H3K36me3 CHIP	GCATCGGAACCATAAGGGGG
	P18 K36me3 R	H3K36me3 CHIP	AAAGTAGAGGCAACGTGGGG
P21	P21 K36me3 F	H3K36me3 CHIP	ATCCCTCCCCAGTTCATTGC
	P21 K36me3 R	H3K36me3 CHIP	GGCTCAACGTTAGTGCCAGG
P27	P27 K36me3 F	H3K36me3 CHIP	TGCCTCTAAAAGCGTTGGATGT
	P27 K36me3 R	H3K36me3 CHIP	TCCACGTCAGTTCCTCAGCC
P57	P57 K36me3 F	H3K36me3 CHIP	TGGGACCGTTCATGTAGCAG
	P57 K36me3 R	H3K36me3 CHIP	CACCTTGGGACCAGTGTACC

Supplementary References

1. Pauklin, S., and Vallier, L. (2013). The cell-cycle state of stem cells determines cell fate propensity. Cell *155*, 135-147. 10.1016/j.cell.2013.08.031.
2. Pauklin, S., Madrigal, P., Bertero, A., and Vallier, L. (2016). Initiation of stem cell differentiation involves cell cycle-dependent regulation of developmental genes by Cyclin D. Genes Dev *30*, 421-433. 10.1101/gad.271452.115.
3. Vallier, L., Touboul, T., Brown, S., Cho, C., Bilican, B., Alexander, M., Cedervall, J., Chandran, S., Ahrlund-Richter, L., Weber, A., and Pedersen, R.A. (2009). Signaling pathways controlling pluripotency and early cell fate decisions of human induced pluripotent stem cells. Stem Cells *27*, 2655-2666. 10.1002/stem.199.
4. Krentz, N.A., Nian, C., and Lynn, F.C. (2014). TALEN/CRISPR-mediated eGFP knock-in add-on at the OCT4 locus does not impact differentiation of human embryonic stem cells towards endoderm. PloS one *9*, e114275. 10.1371/journal.pone.0114275.
5. Hockemeyer, D., Wang, H., Kiani, S., Lai, C.S., Gao, Q., Cassady, J.P., Cost, G.J., Zhang, L., Santiago, Y., Miller, J.C., et al. (2011). Genetic engineering of human pluripotent cells using TALE nucleases. Nat Biotechnol *29*, 731-734. 10.1038/nbt.1927.
6. Tsuneyoshi, N., Tan, E.K., Sadasivam, A., Poobalan, Y., Sumi, T., Nakatsuji, N., Suemori, H., and Dunn, N.R. (2012). The SMAD2/3 corepressor SNON maintains pluripotency through selective repression of mesendodermal genes in human ES cells. Genes Dev *26*, 2471-2476. 10.1101/gad.201772.112.
7. Bertero, A., Madrigal, P., Galli, A., Hubner, N.C., Moreno, I., Burks, D., Brown, S., Pedersen, R.A., Gaffney, D., Mendjan, S., et al. (2015). ACTIVIN/nodal signaling and NANOG orchestrate human embryonic stem cell fate decisions by controlling the H3K4me3 chromatin mark. Genes Dev *29*, 702-717. 10.1101/gad.255984.114.
8. Brown, S., Teo, A., Pauklin, S., Hannan, N., Cho, C.H., Lim, B., Vardy, L., Dunn, N.R., Trotter, M., Pedersen, R., and Vallier, L. (2011). ACTIVIN/Nodal signaling controls divergent transcriptional networks in human embryonic stem cells and in endoderm progenitors. Stem Cells *29*, 1176-1185. 10.1002/stem.666.

SUPPLEMENTARY FIGURE LEGENDS

Supplementary Figure 1: Characterising the cell cycle and expression of CDKIs in hPSCs and germ layers. (a) Differentiation of triple-coloured FUCCI hPSCs indicate the increase in G0 phase cells at day 3 of endoderm differentiation. (b) Characterising the expression of CDKIs by Q-PCR by differentiating hPSCs (UD) to endoderm, mesoderm and neuroectoderm for up to 3 days. (c-d) CDKIs p14, p15, p16, p18, p21 and p57 are induced in endoderm-differentiating hPSCs at day 1 that are losing expression of pluripotency factors (c) OCT4 and (d) NANOG. Scale bar, 100μm. (e) Positive control of endoderm, mesoderm and neuroectoderm differentiation at day 3 show the loss of pluripotency factors OCT4, NANOG and SOX2. Neuroectoderm has high expression of SOX2.

Supplementary Figure 2: Characterising the expression of CDKIs in endoderm, mesoderm and neuroectoderm differentiation at day 3. Co-immunofluorescence microscopy of CDKIs with germ layer markers. Scale bar, 100μm.

Supplementary Figure 3: Developmental signalling pathways including ACTIVIN/TGFβ regulate CDKI expression. (a) CDKI and ki67 expression are non-overlapping in endoderm cells. Scale bar, 50μm. (b) EZH2 and SMYD2 knockdown clones in hPSCs. (c-d) EZH2 inhibition decreases H3K27me3 mark on (c) developmental loci and (d) CDKI loci. (e-f) SMYD2 inhibition decreases H3K4me3 mark on (c) developmental loci and (d) CDKI loci. (g) CDKI expression is distinctly regulated by developmental signalling pathways. Cells at day 2 endoderm were incubated with differentiation media lacking either ACTIVIN A, FGF2, BMP4 or PI3K and with the respective pathway inhibitors. Significant differences compared to OE GFP compared to AFLyB+CHIR sample and calculated by t-test are marked. (h) Concentration dependent induction of CDKIs by ACTIVIN A. Cells were differentiated to endoderm with 10ng/μl or 100ng/μl ACTIVIN A and analysed after

2 days by Q-PCR. Significant differences compared to UD calculated by t-test are marked. (i) Concentration dependent induction of p15 by BMP4. Cells were differentiated to endoderm with 10ng/µl or 100ng/µl BMP4 and analysed after 2 days by Q-PCR. Significant differences compared to UD calculated by t-test are marked. (j) ACTIVIN A and BMP4 regulate CDKI expression. Cells were differentiated to endoderm with different concentration of ACTIVIN A and BMP4, and analysed after 2 days by Q-PCR. (k) SMAD2/3 bind to CDKI loci in pluripotent cells and during endoderm differentiation. SMAD2/3 CHIP was performed at various time points during endoderm differentiation. Significant differences compared to UD SMAD2/3 ChIP calculated by two-way ANOVA are marked. (l) SMAD3 induces the expression of CDKIs in endoderm cells via their promoter regions. SMAD3 and control GFP expressing constructs were transfected to day 1 endoderm cells and analysed by luciferase signal detection after 48h. SB431542 was added 24h before luciferase analysis. Significant differences compared to OE GFP calculated by t-test are marked.

Supplementary Figure 4: The cooperation of EZH2 and SNON with SMAD2/3 and pluripotency transcription factors in hPSCs. (a-b) β-catenin, JUN, JUND, STAT3 and SMAD1 bind to CDKI loci in human pluripotent cells and differentiating hESCs. * marks transcription factor binding peaks. (c-d) OCT4 and NANOG form a complex with SMAD2/3 on CDKI loci in hPSCs. Sequential ChIP of (c) SMAD2/3 and OCT4 or (d) SMAD2/3 and NANOG in hPSC was performed and analysed by Q-PCR. Significant differences compared to IgG/SMAD2/3 sequential ChIP sample calculated by t-test are marked. (e) NANOG knockdown results in reduced SMAD2/3 and SNON binding on CDKI loci. ChIP of NANOG, SMAD2/3 and SNON was performed in Scramble and NANOG KD cells to test their presence on the regions uncovered by genome-wide SMAD2/3 and NANOG ChIP-seq experiments. Significant differences calculated by t-test are marked. NS – not significant. (f)

NANOG knockdown causes a decrease in repressive bivalent mark H3K27me3 on p15, p18, p21 and p57 loci. Scramble and NANOG KD cells were analysed by ChIP-QPCR of H3K4me3 and H3K27me3 marks on CDKI loci. Significant differences calculated by two-way ANOVA are marked. (g) ChIP-qPCR positive control loci OCT4 and NANOG showing the binding of OCT4, NANOG, SMAD2/3 but not SNON and EZH2. (h) SNON binding to CDKI loci is reduced by inhibiting ACTIVIN/TGFβ signalling with SB431542. (i) The recruitment of EZH2 depends on SMAD2/3 on CDKI loci. (j) H3K27me3 deposition on CDKI loci is regulated by SMAD2/3. (k-l) EZH2 and SNON effects can be decoupled. (k) CDKI expression and (l) endoderm marker expression is additively increased upon SNON KD and EZH2 inhibtion. (m) OCT4, NANOG, SMAD2/3, SNON and EZH2 regulate bivalent histone marks on CDKI loci shown by sequential ChIP-qPCR of H3K4me3 and H3K27me3. Statistical analysis was performed by 2-way ANOVA with multiple comparisons with Tukey correction and **** marks adjusted P-value <0.0001, *** is adjusted P-value <0.001, ** is adjusted P-value <0.01, * is adjusted P-value <0.05.

Supplementary Figure 5: SMAD2/3 binds to EZH2 in hPSCs and SMYD2 in endoderm. (a-d) SMAD2/3 switches a complex with EZH2 to SMYD2 on CDKI loci during differentiation. Sequential ChIP of (a-b) SMAD2/3 and EZH2 or vice versa in hESCs, and (c-d) SMAD2/3 and SMYD2 or vice versa in endoderm cells was performed and analysed by Q-PCR. Significant differences compared to IgG sequential ChIP sample calculated by t-test are marked. and **** marks adjusted P-value <0.0001, *** is adjusted P-value <0.001, ** is adjusted P-value <0.01, * is adjusted P-value <0.05. (e) EOMES knockdown in endoderm causes the loss of p15 and p57 expression. Immunostaining of EOMES and p15 or p57 in Scramble and EOMES KD cells differentiated to endoderm for 2 days. Scale bar, 20μm.

Supplementary Figure 6: Knockdown of G1 phase specific inhibitors p15, p18 and p57 alters the efficiency of endoderm differentiation while p21 knockdown reduces neuroectoderm differentiation. (a) Graphics of generating stable CDKI knockdown cell lines in hPSCs. Relative expression of CDKI protein in knockdown clones compared to Scramble cells by western blotting. (b) Knockdown efficiencies of CDKIs assessed by Q-PCR analysis. Significant differences compared to Scramble shRNA calculated by t-test are marked. (c) Overexpression of CDKI shRNA does not disturb pluripotency and self-renewal of hPSCs except for p27. Representative colonies for each CDKI KD cell line. (d) Relative number of colonies derived during CDKI knockdown in pluripotent cells shows a reduction of p27 KD. Significant differences compared to Scramble shRNA calculated by t-test are marked. (e) Knockdown of cell cycle inhibitors alters the background differentiation of hPSCs. Differentiation markers were analysed by their relative mRNA expression in knockdown cells and Scramble cells. Significant differences compared to Scramble shRNA calculated by t-test are marked. (f-i) CDKI expression changes the background differentiation of cells in a germ layer specific manner. Flow cytometry analysis of (f) endoderm, (g) mesoderm, (h) neuroectoderm markers and (i) pluripotency markers in CDKI KD lines. Significant differences compared to Scramble shRNA calculated by t-test are marked. **** marks adjusted P-value <0.0001, *** is adjusted P-value <0.001, ** is adjusted P-value <0.01, * is adjusted P-value <0.05

Supplementary Figure 7: CDKIs alter the efficiency of germ layer differentiation. (i) p16 alters mesoderm differentiation. Q-PCR analysis of mesoderm markers in CDKI KD cells differentiated to mesoderm. Significant differences compared to Scramble shRNA calculated by t-test are marked. (b) Schematic overview of CDKI effects on germ layer specification. (c) Summary of germ layer specific functions of CDKIs. (d-e) CDKI knockdown results in germ layer-specific effects on

differentiation. Flow cytometry analysis of differentiation markers during (c) endoderm and (d) neuroectoderm differentiation. Significant differences compared to differentiated Scramble shRNA sample calculated by t-test are marked. (f) p15, p18 and p57 regulate endoderm differentiation. The efficiency of endoderm differentiation were analysed by relative expression of NANOG and SOX2 protein by flow cytometry. Significant differences compared to Scramble shRNA calculated by t-test are marked. (g) p21 regulates neuroectoderm differentiation. Marker expression was analysed by flow cytometry. Significant differences compared to Scramble shRNA and calculated by t-test are marked. Statistical analysis was performed by 2-way ANOVA with multiple comparisons with Tukey correction and **** marks adjusted P-value <0.0001, *** is adjusted P-value <0.001, ** is adjusted P-value <0.01, * is adjusted P-value <0.05. (h-i) Teratoma assays of CDKI knockdown in hESCs indicate changed differentiation efficiencies upon CDKI knockdown. (h) Schematic overview of teratoma assays. (i) Representative section of teratomas from Scramble, p18 KD, p21 KD and p57 KD cells. Undifferentiated cells were injected into the testes and allowed to form teratomas for 3 months before histological analysis.

Supplementary Figure 8: Overexpression of CDKIs alters germ layer specification. (a) Confirmation of CDKI overexpression clones. Schematic depiction of generating stable overexpressing hPSCs together with cell cycle inhibitor protein expression. (b) The number of colonies derived by stable expression is severely reduced in cells overexpressing CDKIs compared to OE GFP cells. Significant differences compared to OE GFP calculated by t-test are marked. (c) Relative levels of CDKI analysed by mRNA overexpression. Significant differences compared to OE GFP calculated by t-test are marked. (d) p18, p15 and p57 overexpression in hPSCs results in the lengthening of G1 phase. Cell cycle was analysed by EdU incorporation and flow cytometry. (e) The G1 phase is extended in Cyclin D double knockdown cells. Cell cycle analysed by EdU

incorporation and flow cytometry showed that Cyclin D double knockdown mimics overexpression of CDKIs in its effects on cell cycle and endoderm differentiation. (f-i) Overexpression of cell cycle inhibitors in hPSCs induces differentiation toward distinct germ layers. Flow cytometry analysis of (f) endoderm, (g) mesoderm, (h) neuroectoderm or (i) pluripotency markers. Significant differences compared to OE GFP calculated by t-test are marked.

Supplementary Figure 9: Overexpression of p15, p18 and p57 promotes endoderm differentiation. (a) Expression of p15, p18 and p57 enhances endoderm specification. Immunostaining of endoderm marker Sox17 and pluripotency marker Tra-1-60 in endoderm differentiation conditions which have a 10-fold reduced Activin A concentration at 10 ng/µl. (b-e) Changes in marker expression during endoderm and neuroectoderm differentiation in CDKI expressing cells compared to cells with an empty vector. Flow cytometry analysis of markers upon (b, d) endoderm and (c, e) neuroectoderm differentiation. Significant differences compared to OE GFP calculated by t-test are marked. (f-h) Effects of p27 inducible overexpression on (f) endoderm markers, (g) cell cycle, and (h) SMAD2/3 dependent promoter-luciferase activity. (i) Co-immunofluorescence microscopy of OCT4 and SOX17 or NANOG and SOX17 in CDKI OE hPSCs indicates increased propensity to induce SOX17 and lose the expression of pluripotency markers. Scale bar, 100μm.

Supplementary Figure 10: CDKI OE supports endoderm differentiation. (a) The binding of SMAD2/3 to its endodermal target loci in late G1 phase is increased by p15 and p18 expression. FUCCI-hPSCs were transfected with CDKI expressing constructs, sorted after 48 hours to early G1 and late G1 phase, followed by SMAD2/3 CHIP. Significant differences compared to OE pTP6

vector transfection calculated by t-test are marked. (b-d) SMAD2/3 binding to its target loci during endoderm differentiation is disrupted by p15, p18 and p57 knockdown. SMAD2/3 CHIP was performed in differentiating cells at various time points and Smad2/3 binding was analysed on (b) Mixl1, (c) EOMES and (d) SOX17 promoters. Significant differences compared to Scramble shRNA for each time point and calculated by two-way ANOVA are marked. (e) Graphical depiction of subcellular fractionation in CDKI OE and KD cells and the abundance of chromatin marker Histone H3 and cytoplasmic marker actin. (f) CDKIs control SMAD2/3 transcriptional activity on SOX17 promoter region. Cells were co-transfected with a combination of CDKI constructs and a luciferase construct under the regulation of SOX17 promoter region. Significant differences compared to OE SMAD3 calculated by t-test are marked.

Supplementary Figure 11: Positive feedback loops between SMAD2/3 and CDKIs drive stepwise specification toward definitive endoderm. (a) SMAD2/3 transcriptional activity is elevated by CDKIs due to the lengthening of G1 phase. The relative luciferase signals were analysed in each cell cycle phase in cells transfected with SBE4-luc promoter construct and with p18 overexpression construct. Significant differences compared to empty vector transfected endoderm sample at each cell cycle phase calculated by two-way ANOVA are marked. (b) SMAD2/3 binding to endoderm loci is extended through the whole lengthened G1 phase. SMAD2/3 CHIP was performed on Day 1 endoderm cells transfected with p18 or pluripotent FUCCI-hESCs that were sorted into distinct cell cycle phases. Significant differences compared to empty vector transfected endoderm sample at each cell cycle phase and calculated by t-test are marked. All data are shown as mean+/-s.d. (n=3). (c) SMAD2/3 binding to CDKI loci is extended throughout the whole lengthened G1 phase. Smad2/3 CHIP was performed on pluripotent FUCCI-hESCs and day 1 endoderm cells transfected with p18 sorted into distinct cell cycle phases.

ACTIVIN/NODAL and cell cycle inhibitors drives the lengthening of G1 phase, which in turn allows for a prolonged activity of SMAD2/3 during differentiation that is necessary for definitive endoderm formation. Significant differences compared to endoderm cells with OE pTP6 vector and calculated by two-way ANOVA are marked. (d) Schematic depiction of the circuitry of SMAD2/3, developmental master regulators and CDKIs that directs lineage specification. NANOG-SMAD2/3-SNON keep p18 and p15 in a poised state for rapid activation. Upon the presence of differentiation signals, NANOG and SNON are removed from p18 and p15 loci due to degradation, which allows SMAD2/3 to activate their expression, thus forming a positive regulatory loop. This involves CDK4/6 inhibition by p18 and p15, which in turn hyper-activates SMAD2/3 dependent induction of developmental master regulators such as EOMES. EOMES in turn cooperates with SMAD2/3 to further induce CDKI expression, that drive G1 phase lengthening and the stepwise activation of downstream gene circuitries for definitive endoderm identity. (e) Schematic depiction of the circuitry of SMAD2/3, developmental master regulators and CDKIs that directs lineage specification. NANOG-SMAD2/3-EZH2-SNON keep p18 and p15 in a poised state for rapid activation. Upon the presence of differentiation signals, NANOG and SNON are removed from p18 and p15 loci due to degradation, which allows SMAD2/3 to activate their expression, thus forming a positive regulatory loop. This involves CDK4/6 inhibition by p18 and p15, which in turn hyper-activates SMAD2/3 dependent induction of developmental master regulators such as EOMES. EOMES in turn cooperates with SMAD2/3 to further induce CDKI expression, that drive G1 phase lengthening and the stepwise activation of downstream gene circuitries for definitive endoderm identity. (f) Effects of EZH2 and SMYD2 inhibitors on pancreatic islet cell type specification.

Supplementary Figure 1

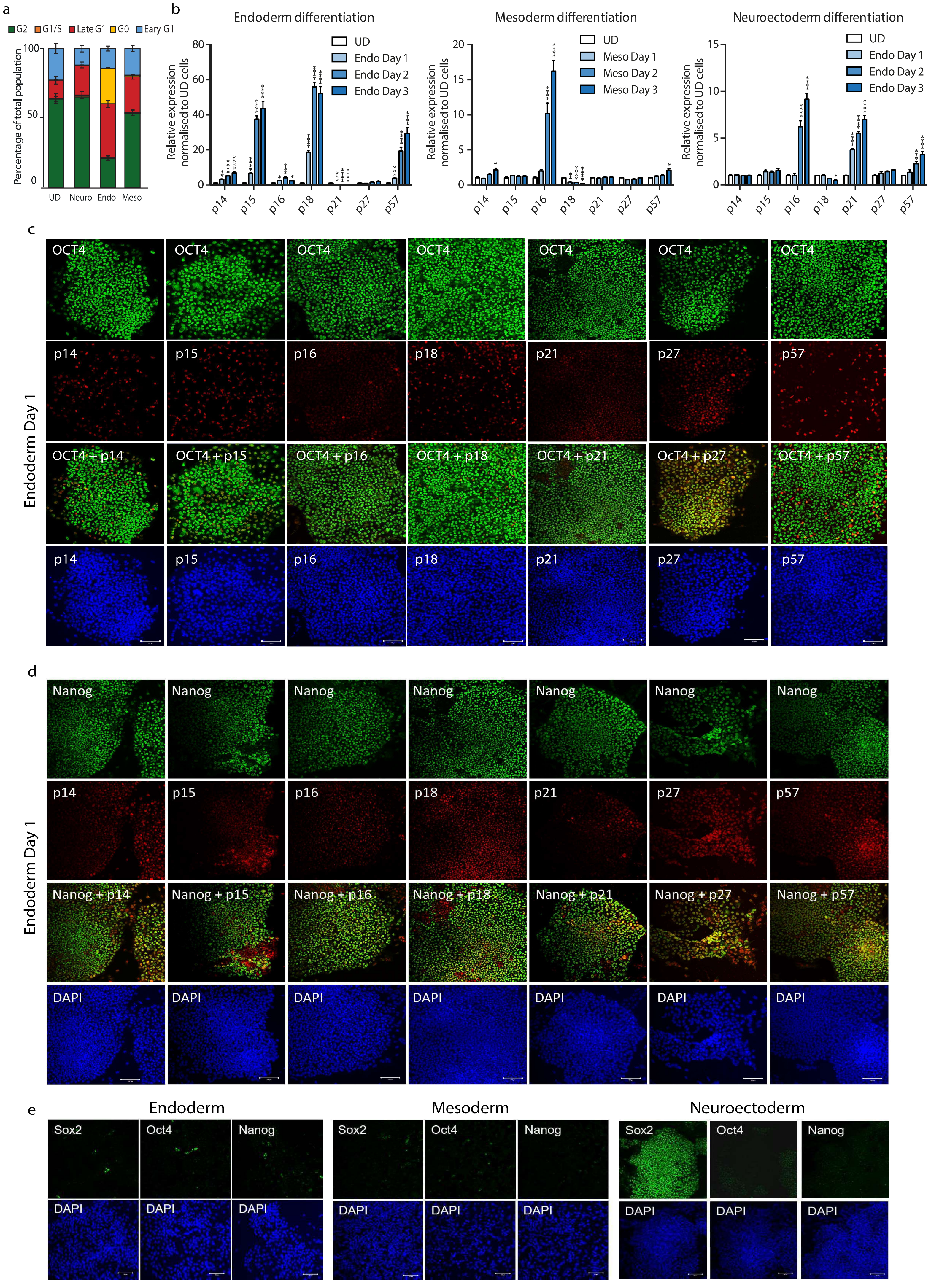

Supplementary Figure 2

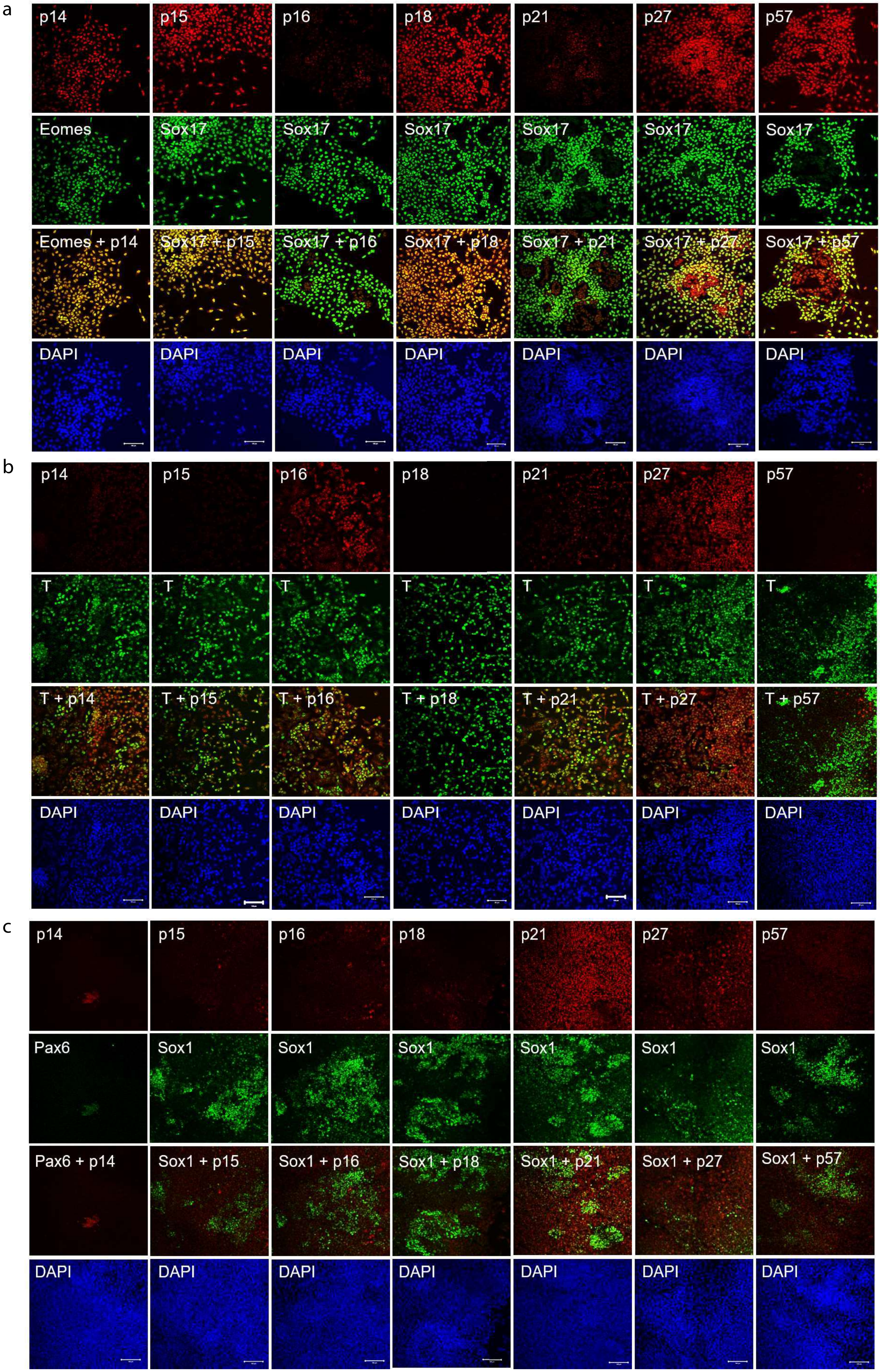

Supplementary Figure 3

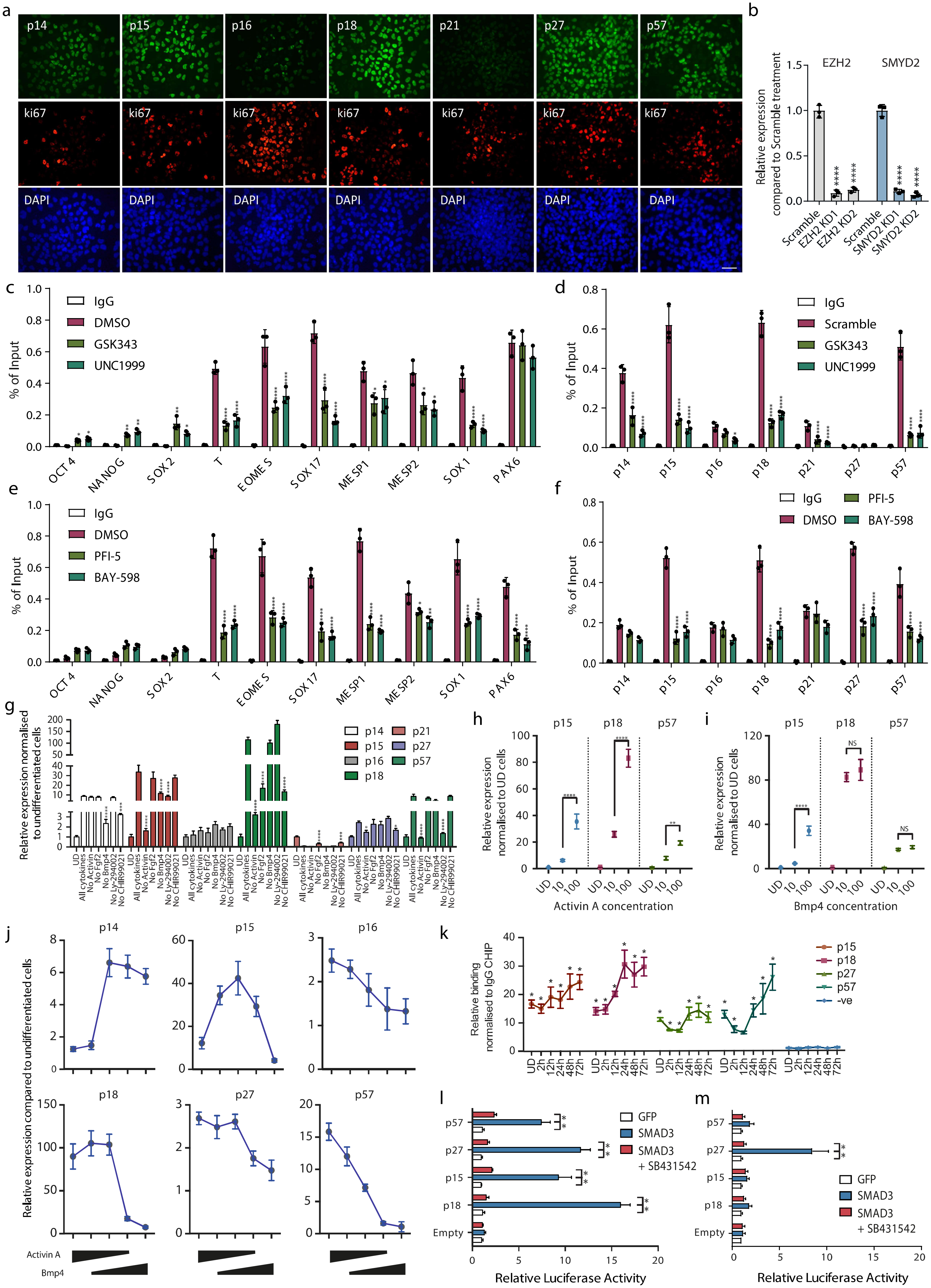

Supplementary Figure 4

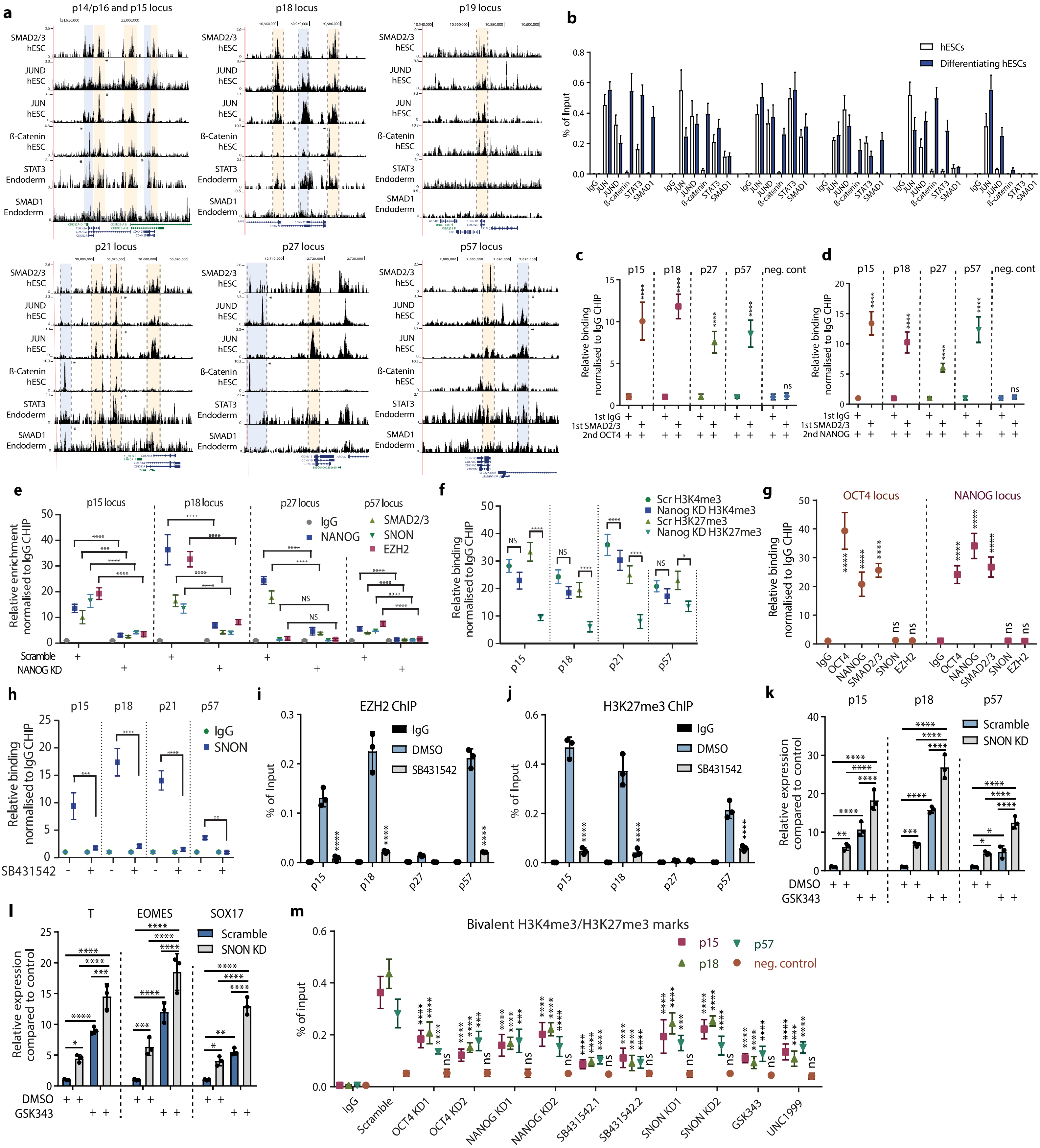

Supplementary Figure 5

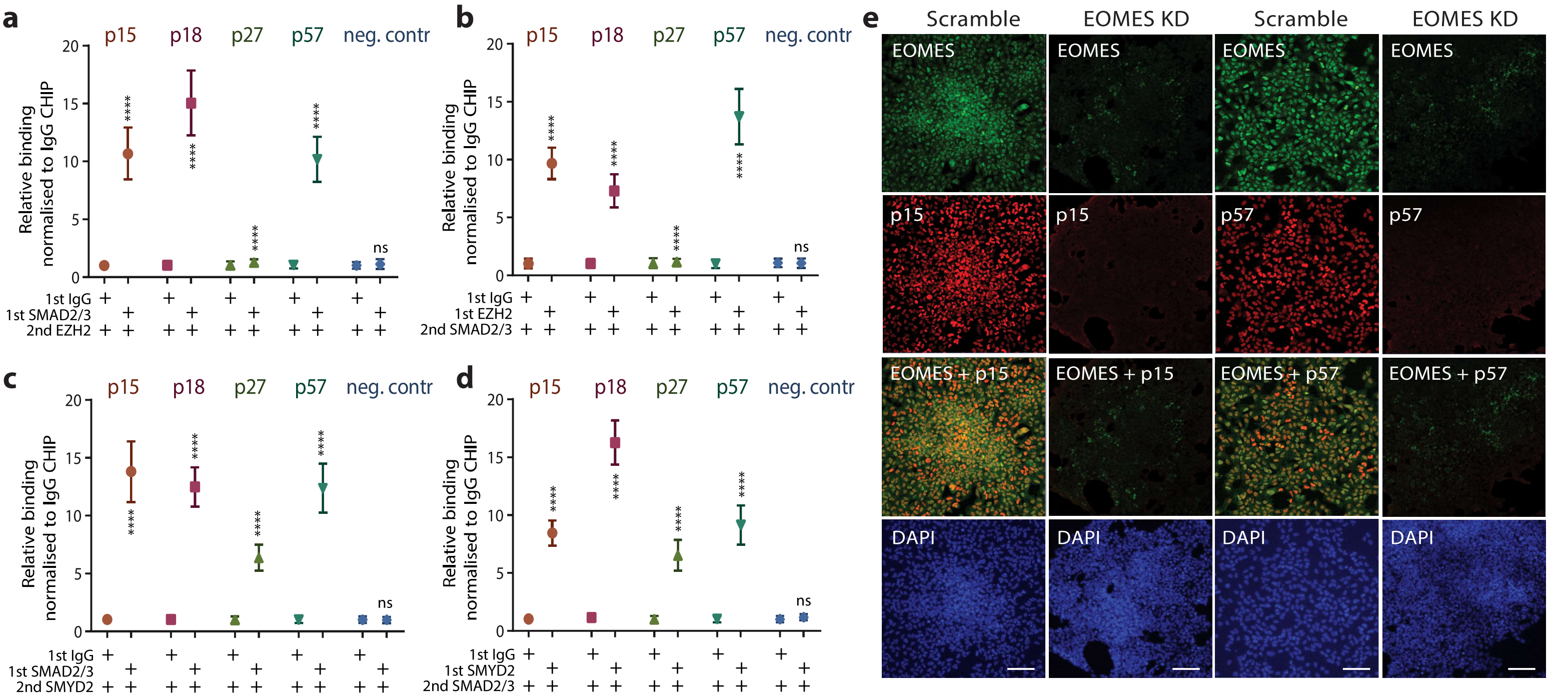

Supplementary Figure 6

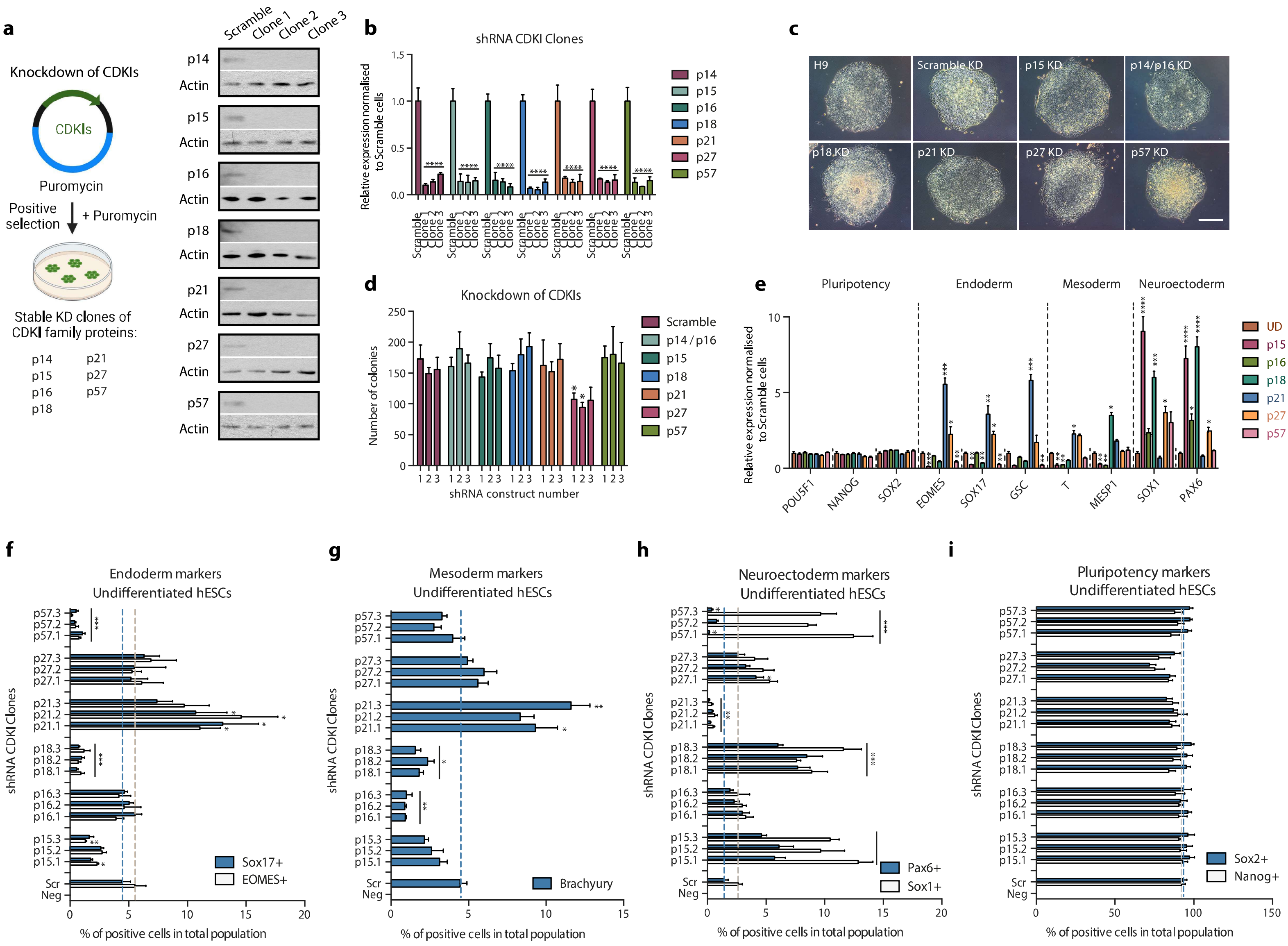

Supplementary Figure 7

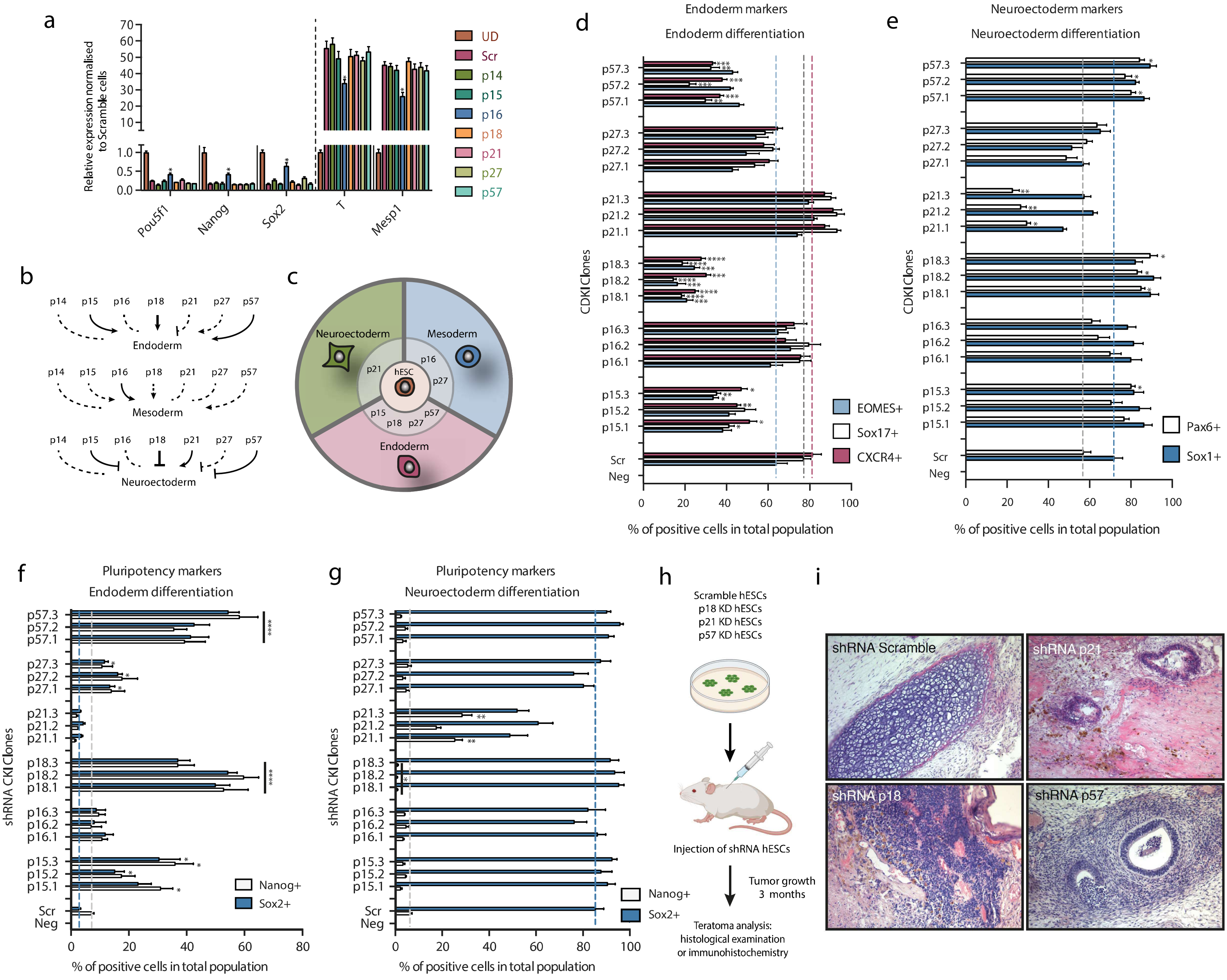

Supplementary Figure 8

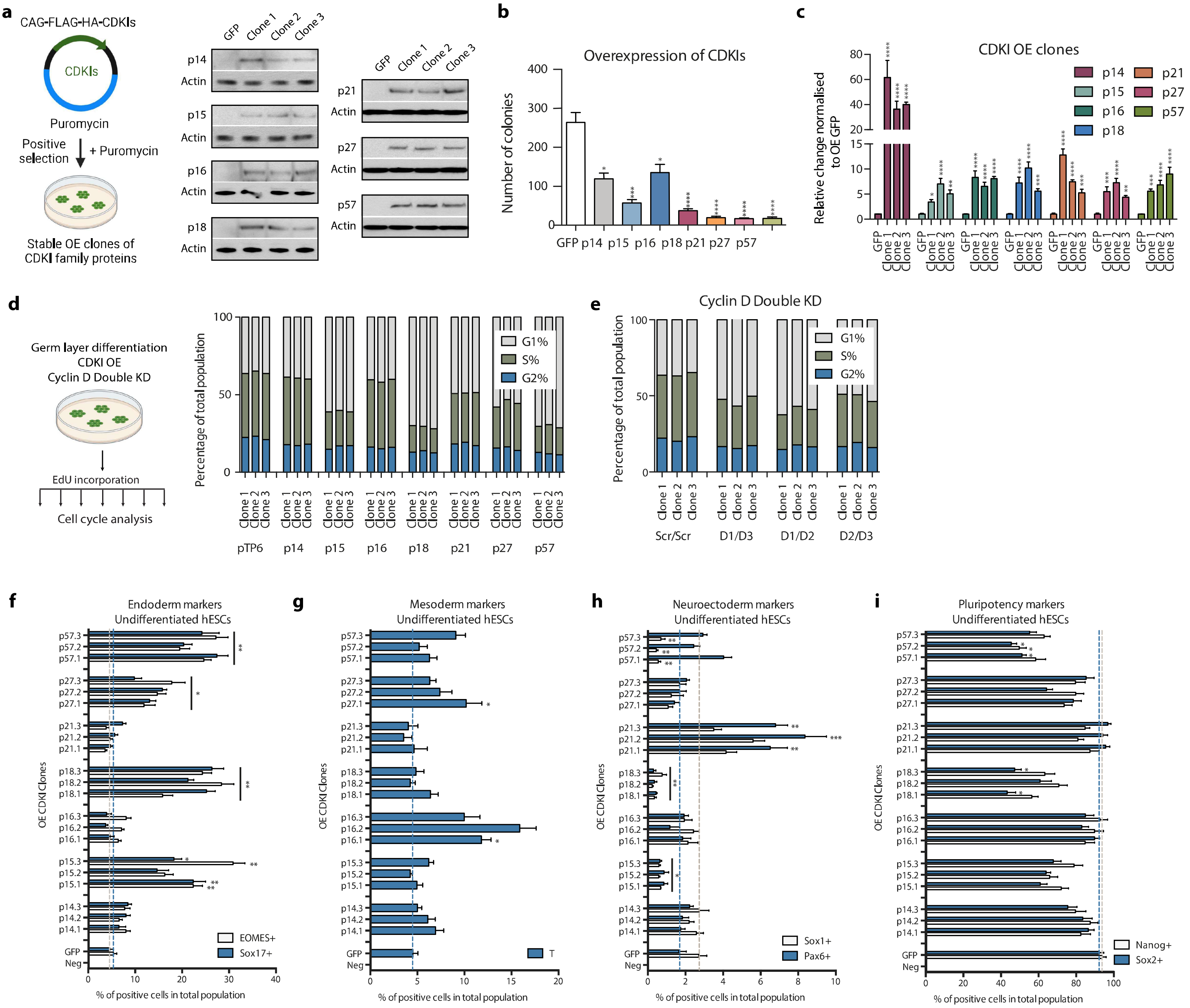

Supplementary Figure 9

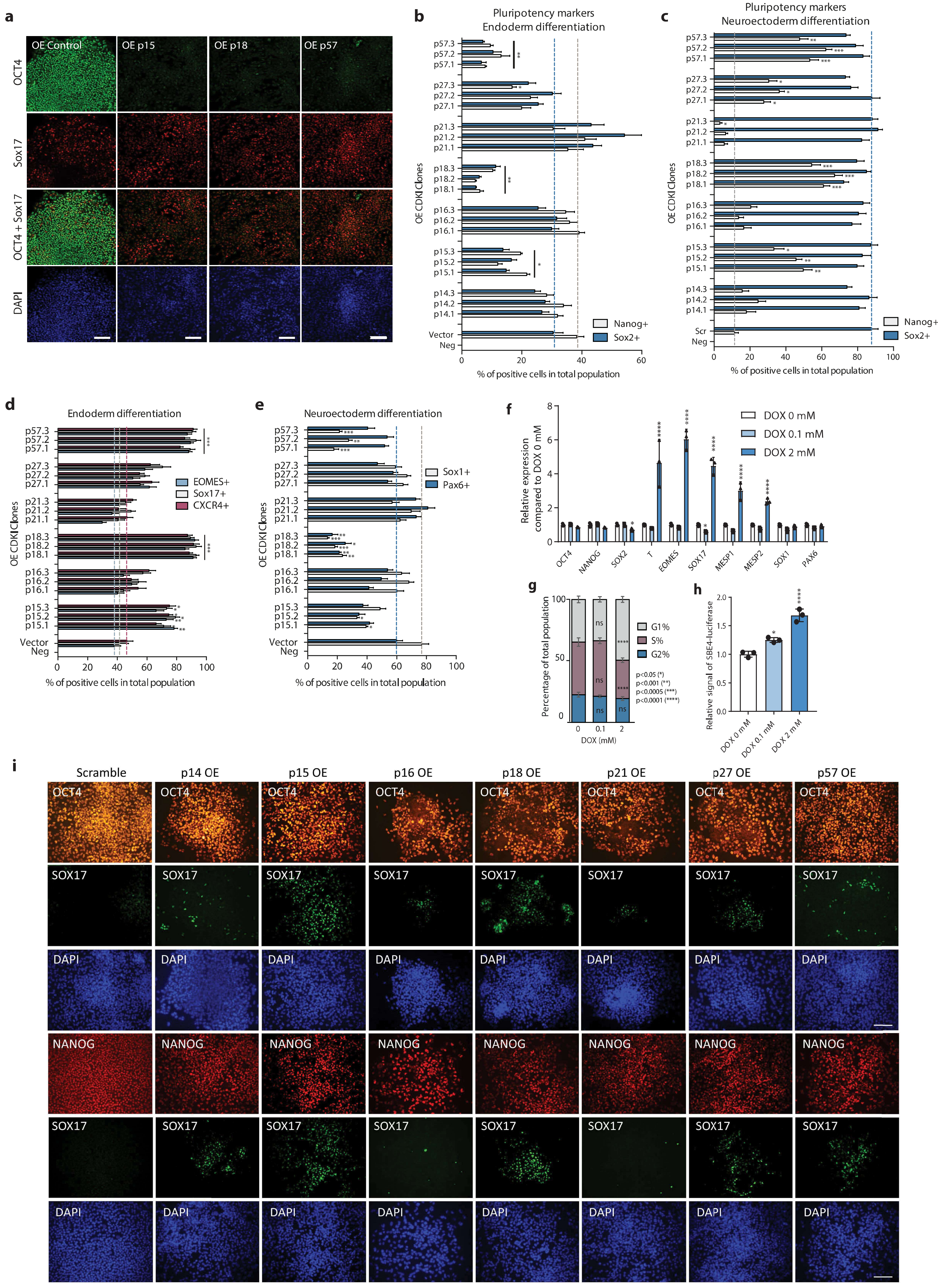

Supplementary Figure 10

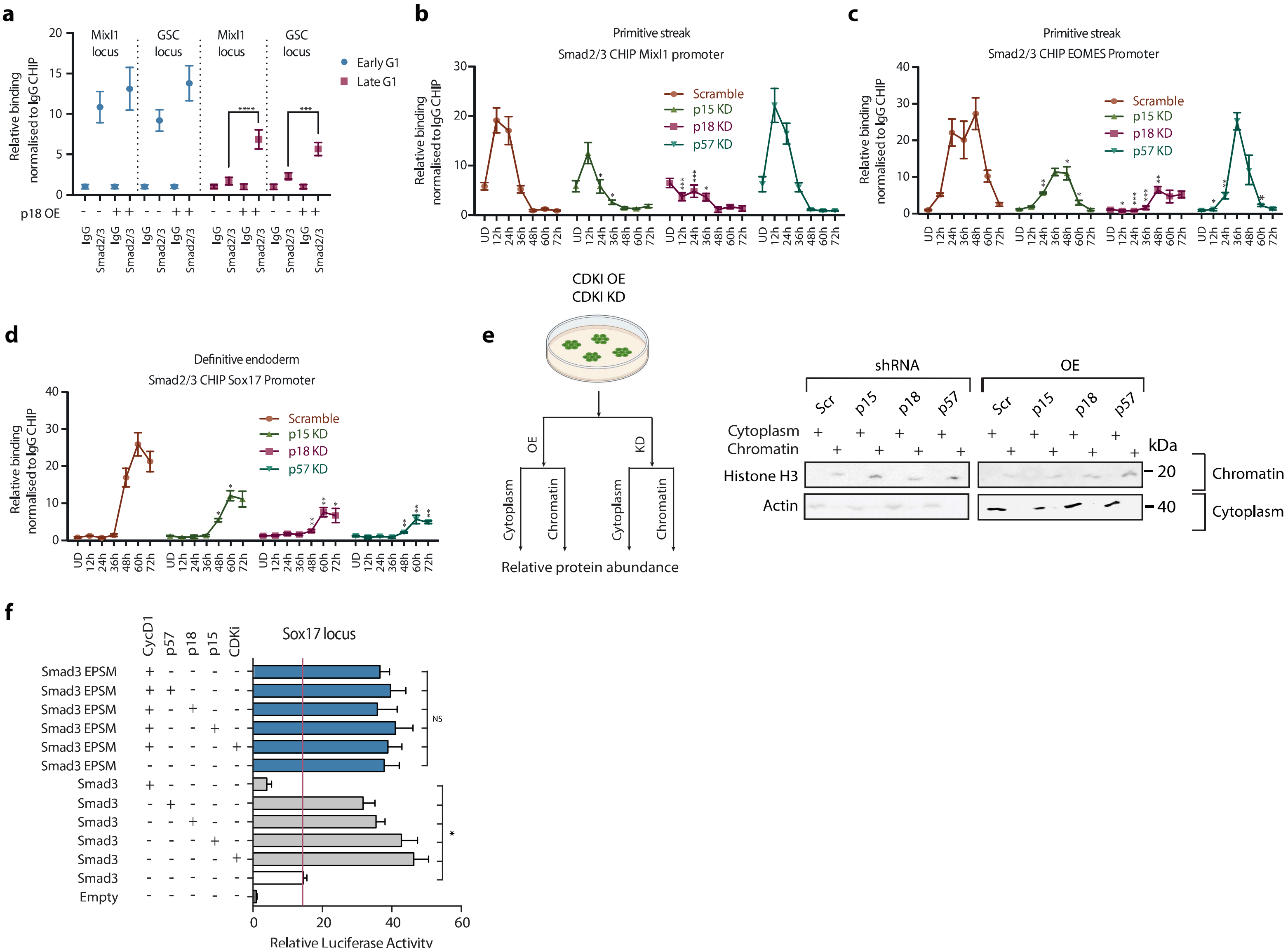

Supplementary Figure 11

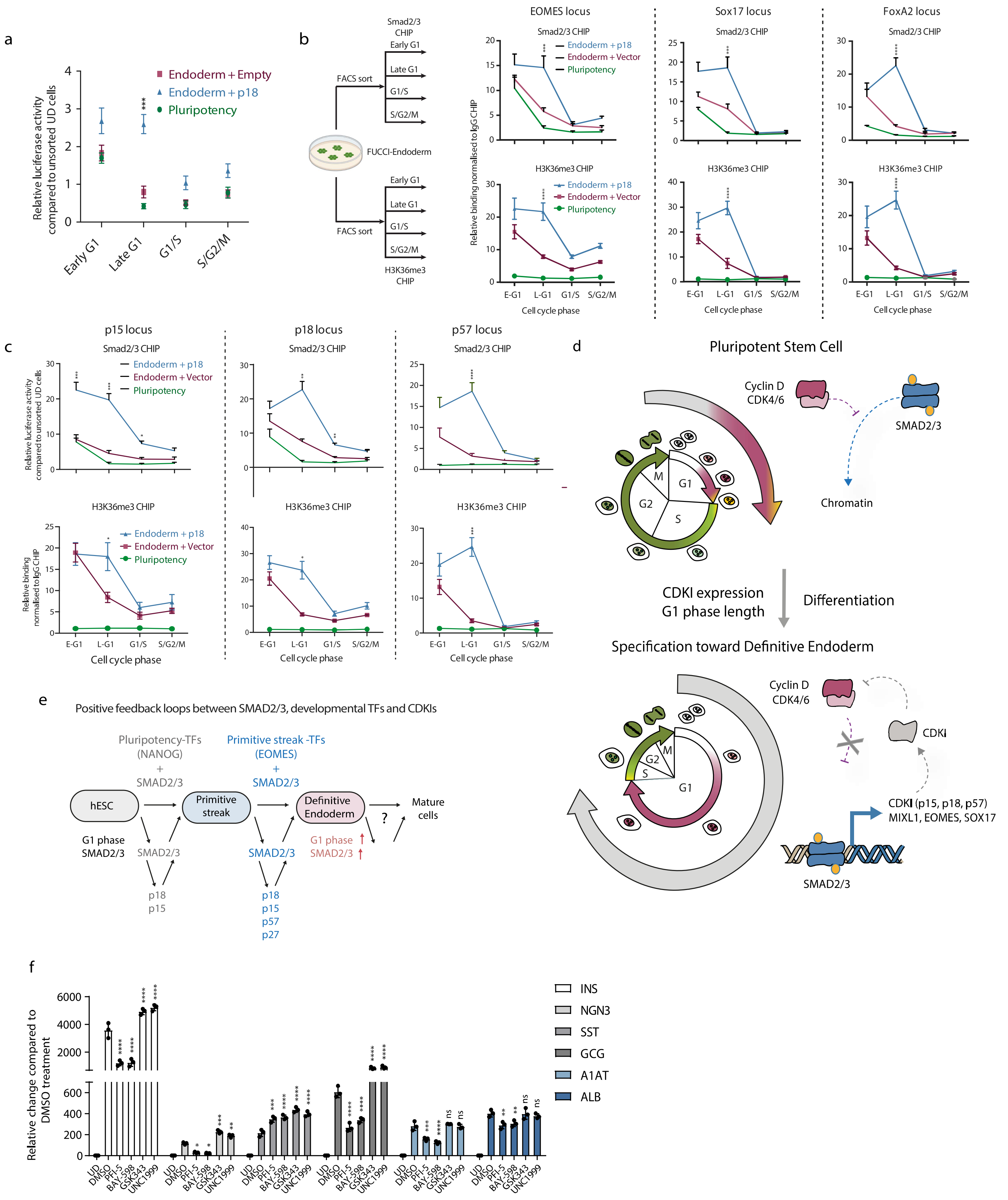

Supplement: pwae031_suppl_Supplementary_Tables_S1-S6_Figures_S1-S11 [file pwae031_suppl_supplementary_tables_s1-s6_figures_s1-s11.pdf]
